# MicroRNA-1205 Regulation of FRYL in Prostate Cancer

**DOI:** 10.3389/fcell.2021.647485

**Published:** 2021-07-27

**Authors:** Michelle Naidoo, Fayola Levine, Tamara Gillot, Akintunde T. Orunmuyi, E. Oluwabunmi Olapade-Olaopa, Thahmina Ali, Konstantinos Krampis, Chun Pan, Princesca Dorsaint, Andrea Sboner, Olorunseun O. Ogunwobi

**Affiliations:** ^1^Department of Biological Sciences, Hunter College of the City University of New York, New York, NY, United States; ^2^Department of Biology and Biochemistry, The Graduate Center of the City University of New York, New York, NY, United States; ^3^Department of Radiation Oncology, College of Medicine, University of Ibadan, Ibadan, Nigeria; ^4^Department of Surgery, College of Medicine, University of Ibadan, Ibadan, Nigeria; ^5^Department of Mathematics and Statistics, Hunter College of the City University of New York, New York, NY, United States; ^6^Englander Institute for Precision Medicine, Weill Cornell Medicine, New York, NY, United States; ^7^Joan and Sanford I. Weill Department of Medicine, Weill Cornell Medicine, New York, NY, United States

**Keywords:** prostate cancer, FRYL, microRNA-1205, neuroendocrine prostate cancer, neuroendocrine differentiation

## Abstract

High mortality rates of prostate cancer (PCa) are associated with metastatic castration-resistant prostate cancer (CRPC) due to the maintenance of androgen receptor (AR) signaling despite androgen deprivation therapies (ADTs). The 8q24 chromosomal locus is a region of very high PCa susceptibility that carries genetic variants associated with high risk of PCa incidence. This region also carries frequent amplifications of the PVT1 gene, a non-protein coding gene that encodes a cluster of microRNAs including, microRNA-1205 (miR-1205), which are largely understudied. Herein, we demonstrate that miR-1205 is underexpressed in PCa cells and tissues and suppresses CRPC tumors *in vivo*. To characterize the molecular pathway, we identified and validated fry-like (FRYL) as a direct molecular target of miR-1205 and observed its overexpression in PCa cells and tissues. FRYL is predicted to regulate dendritic branching, which led to the investigation of FRYL in neuroendocrine PCa (NEPC). Resistance toward ADT leads to the progression of treatment related NEPC often characterized by PCa neuroendocrine differentiation (NED), however, this mechanism is poorly understood. Underexpression of miR-1205 is observed when NED is induced *in vitro* and inhibition of miR-1205 leads to increased expression of NED markers. However, while FRYL is overexpressed during NED, FRYL knockdown did not reduce NED, therefore revealing that miR-1205 induces NED independently of FRYL.

## Introduction

Prostate cancer (PCa) was the second most diagnosed and deadliest cancer in 2020 with an estimate of 209,512 cases and 32,438 deaths in the United States according to GLOBOCAN2020 (Ferlay et al., 2020). Although men diagnosed with local and regional PCa have 100% 5-years period survival rate, patients diagnosed with PCa at the distant stage have a relative 30% 5-years survival rate, indicating that the spread of the disease is extremely lethal (Howlander et al., 2017). Although the androgen receptor (AR) is not the only driver of prostate carcinogenesis, almost 80–90% of prostate cancers are dependent on androgenic activity via AR signaling (Denis and Griffiths, 2000). Therefore, androgen deprivation therapy is critical for patients that display indications of high-risk localized or metastatic PCa and is currently the most effective treatment that significantly improves survival rates (Huggins, 1967; Huggins and Hodges, 1972; Harris et al., 2009). However, 10–20% of patients develop castration resistant PCa (CRPC) due to failure of tumor regression upon treatment, in which 33% with CRPC develop metastatic CRPC (mCRPC) within 2 years of diagnosis (Morgan et al., 2010; Kirby et al., 2011; Hirst et al., 2012). While several next generation hormone treatments, such as abiraterone acetate and enzalutamide, enhances survival rates for men diagnosed with mCRPC, approximately 20–40% of patients do not respond to treatments and those who initially respond to treatments may acquire secondary resistance (Mostaghel et al., 2011; Antonarakis et al., 2014). Moreover, as PCa is a very heterogeneous disease, some patients also acquire rare PCa subtypes after ADT relapse, such as neuroendocrine prostate cancer (NEPC), that are characterized as AR-negative and survive with complete independence of AR signaling. More recently, NEPC has been observed in 15–20% of patients with prostate adenocarcinoma who received chemotherapy, including treatments with abiraterone and enzalutamide (Conteduca et al., 2019). The transformation from prostate adenocarcinoma to NEPC as a consequence of androgen deprivation therapies is clinically termed treatment-related NEPC (t-NEPC) (Ito et al., 2001; Hirano et al., 2004; Aparicio et al., 2011; Robinson et al., 2015). Although NEPC is rare (< 2% incidence in the United States), men who develop NEPC or t-NEPC have very poor prognosis because of lack of targeted therapies and insufficiently identified biomarkers (Beltran et al., 2011). Resistance toward ADTs creates a new challenge for treating men with CRPC and better understanding of these mechanisms will provide knowledge on how to address PCa heterogeneity.

Genome wide association studies (GWAS) has identified over 70 genetic variants that are associated with high risk for developing PCa (Benafif et al., 2018). The 8q24 chromosomal region was one of the first regions to be identified and is considered to be the most important PCa susceptibility locus (Yeager et al., 2007). This region is commonly known to be a “gene desert” due to the lack of protein coding genes (Ghoussaini et al., 2008; Wasserman et al., 2010). Within the 8q24 chromosomal region lies the PVT1 gene, which encodes a series of alternatively spliced transcripts along with a cluster of microRNAs (–1204, –1205, –1206, –1207-5p, –1207-3p, and –1208) (Cory et al., 1985; Huppi et al., 2008; Colombo et al., 2015). PVT1 is amplified in many cancers and has been reported to function as a microRNA sponge ultimately inducing proliferation and suppressing apoptosis in cancer cells (Guan et al., 2007; Ding et al., 2014; Paci et al., 2014; Wang et al., 2014; Yang et al., 2014; Ogunwobi and Kumar, 2019). Moreover, our lab has demonstrated the potential role of PVT1 as a biomarker in aggressive prostate cancer (Ilboudo et al., 2015). However, the importance of PVT1-encoded microRNAs are largely understudied. Recent reports have shown the implications of microRNA-1204 in the suppression tumor growth, suggesting a role in oncogenesis (Beck-Engeser et al., 2008; Barsotti et al., 2012). Furthermore, our lab has established the clinical significance of microRNA-1207-3p as a biomarker and putative therapeutic option via a novel regulatory pathway in prostate cancer (Das et al., 2016a). A more in-depth investigation of PVT1-encoded miRNAs will further demonstrate the importance of the 8q24 chromosomal locus in PCa.

Due to the increasing evidence of PVT1-encoded miRNAs in cancer, we were interested in elucidating the role of miR-1205 in cancer. It was previously reported that miR-1205 is expressed at very low levels among cancer cell lines (not including PCa cell lines), questioning its role in tumorigenesis (Beck-Engeser et al., 2008; Huppi et al., 2008). The aim of this study was to examine the role of miR-1205 in PCa. We observed underexpression of miR-1205 and overexpression of Fry-like (FRYL), a putative target of miR-1205, in PCa tissues and cell lines. Moreover, we generated novel synthetic analogs of miR-1205 and discovered that miR-1205 can inhibit castration-resistant prostate cancer growth in mice. Further investigation of miR-1205 tumor suppressive pathway confirmed direct miR-1205:FRYL binding and putative role of this pathway in PCa neuroendocrine differentiation (NED), a phenomenon occurring in resistant PCa subtypes.

## Materials and Methods

### Patient Cohorts

Tissues were collected in compliance with Institutional Ethics Board approved protocol at the City University of New York and University of Ibadan. All experiments were performed in accordance with relevant guidelines and regulations. Informed consent was acquired for all participants. Deidentified frozen tissue was obtained from prostatectomy or transrectal ultrasounded-guided biopsies in patients from University of Ibadan. All tissues were histologically identified as normal prostatic (*n* = 22), benign prostatic hyperplasia (*n* = 42), or prostate cancer tissue (*n* = 26). RNA extraction, cDNA synthesis and RT-qPCR was performed to analyze mRNA expression of miR-1205 and FRYL.

Additionally, FRYL expression was examined in a RNA-seq analysis of prostate cancer and adjacent normal tissue (*n* = 14) of a Chinese population using the galaxy web platform.^1^

Lastly, we looked at the expression levels of FRYL in a Weill Cornell Medicine (WCM) cohort of 29 benign prostate samples, 66 localized prostate cancer (PCa), 73 castration resistance adenocarcinomas (CRPC), and 36 neuroendocrine prostate cancer (NEPC) samples whose transcriptome was profiled by RNA-seq (Berger et al., 2019). Similarly to the original analysis, we aligned all reads against the human genome sequence build hg19^2^ with STAR_2.4.0f1 (Dobin et al., 2013) and used SAMTOOLS v0.1.19 (Li et al., 2009) for sorting and indexing reads. We estimated expression values (FPMKs—fragments per million reads per kilobase of exons) with Cufflinks (2.0.2) (Trapnell et al., 2014) based on GENCODE v19 (Derrien et al., 2012) gene annotation. Since the sequenced samples from the published datasets were processed using different library preps, i.e., poly-A selection or ribosomal depletion, we normalized FPKMs via ComBat (Johnson et al., 2007) from sva bioconductor package (Leek et al., 2012). This way the differences due to the library preparation methods are reduced.

### Cell Culture

Cell culture of human RWPE-1 (normal prostate epithelial cells) (Bello et al., 1997), WPE1-NA22 (indolent PCa epithelial cells) (Webber et al., 2001), MDA PCa 2b (metastatic PCa adenocarcinoma epithelial cells) (Navone et al., 1997), PC-3 (androgen-independent metastatic PCa cells) (Kaighn et al., 1979) and LNCaP (androgen-sensitive PCa cells) (Horoszewicz et al., 1980) were performed as previously described (Das et al., 2016a). 22Rv1 (human androgen-insensitive PCa cells) was purchased from the American Type Culture Collection and cultured in RPMI 1640 supplemented with 10% heat inactivated FBS, and 1% penicillin/streptomycin (Sramkoski et al., 1999). C4-2B (human androgen-insensitive PCa cells) was cultured in DMEM supplemented with 200 ml Ham’s F12, 10% heat-inactivated FBS, 1% penicillin/streptomycin, insulin (5 μg/ml), triiodothyronine (13.65 pg/ml), human apo-transferrin (4.4 μg/ml), d-biotin (0.244 μg/ml), and adenin (12.5 μg/ml). The C4-2B cell line is a well validated and widely recognized model of CRPC obtained from MD Anderson Cancer Center under a materials transfer agreement with Hunter College of The City University of New York (Thalmann et al., 1994).

RPMI 1640 medium containing 10% charcoal-stripped FBS and 1% penicillin/streptomycin was used to differentiate LNCaP cells into neuroendocrine-like PCa cells. Control LNCaP cells were seeded in a 10 cm culture dish and were maintained with RPMI 1640 medium containing 10% heat-inactivated FBS and 1% penicillin/streptomycin. LNCaP and LNCaP-NED cells were cultured for 10 days and pellets were collected for downstream analysis.

### Synthesis of Synthetic miR-1205 Analogs and Control Oligonucleotides

Synthesis of negative control scramble oligonucleotides (NB1) and synthetic miR-1205 analogs (NB1205) was carried out on an automated synthesizer using standard protocols and HPLC-purified. Purified products are stored as lyophilized powder at –20°C until use.

### Transfections of Oligonucleotides and siRNAs

Transfection of miR-1205 mimic, miR-1205 inhibitor, NB1 and NB1205 were performed as previously described (Das et al., 2016a). MicroRNA-1205 mimic (MISSION microRNA Mimic—hsa-miR-1205, HMI0063), its inhibitor (MISSION Synthetic microRNA Inhibitor—hsa-miR-1205, HSTUD0063) and FRYL siRNA were purchased from Sigma-Aldrich. Transfection of siRNAs were performed as described (Das et al., 2016b). Negative control scramble siRNAs which do not lead to specific degradation of any known mRNA were used as negative controls (Das et al., 2016a).

### RNA Isolation and Quantitative Real-Time Polymerase Chain Reaction (qRT-PCR) Analysis

RNA isolation, cDNA synthesis, and qRT-PCR were performed as previously described (Das et al., 2016a). All experiments were performed three times in quadruplicates. GAPDH and FRYL human primers were purchased from Sigma-Aldrich (St. Louis, MO, United States). MiR-1205 and RNU6B primers were purchased from Qiagen (Hilden, Germany). The expression of genes was normalized to the expression of GAPDH or U6 genes. Primer sequences for FRYL: Forward: 5′-AGC ATT GTA GCT GTT GGT TTGT; Reverse: 5′-AGG GCA ATT CAA GAA GGT AACA.

### Luciferase Report Assay

Human FRYL (HmiT008154-MT06) and miRNA target clone control vector for pEZX-MT06 were purchased from Genecopoeia^TM^, Rockville, MD, United States. Cells were seeded in a 24 well plate and co-transfected with either a negative control oligonucleotide (MISSION Negative Control 1, NSTUD001), miR-1205 mimic, or miR-1205 mutant and/or miRNA target clone control vector or FRYL 3′UTR target clone vector. Lysates were analyzed using the Luc-Pair^TM^ Duo-Luciferase Assay kit 2.0 (LF001, Genecopoeia). Luminescence was detected using the SpectraMax i3x multi-mode detection platform.

### Western Blotting

Western blotting was performed as described previously (Das et al., 2016a). Primary antibodies against human FRYL: PA5-56644 (Thermo Fisher Scientific, United States), Aurora A: 14475 (Cell Signaling), NSE: NB200-421 (Novus Biologicals), human GAPDH: 5174 (Cell Signaling) and alpha tubulin: sc-32293 (Santa Cruz Biotechnology). Secondary antibodies used were either against mouse or rabbit (LI-COR, St. Louis, MO, United States).

### RNA Pulldown

RNA pulldown was similarly performed as previously described (Das et al., 2016a). The DynaBeads Myone Streptavidin C1 (Ref: 65001, Thermo Fisher Scientific) were washed three times with bead wash buffer (5 mM Tris-Cl pH 7.5, 0.5 mM EDTA, and 1M NaCl) and blocked overnight (1 μg/μl BSA and 1 μg/μl yeast tRNA). Cells transfected with 7 nM of the miR-1205 or scramble biotinylated duplex were harvested and lysed after 24 h. The lysate and beads were mixed and incubated overnight at 4°C. The following day, beads were washed and target mRNA associated with the duplex was isolated using TRIzol (Ref:15596026, Thermo Fisher Scientific). To determine enrichment of target mRNA, qRT-PCR analysis were performed to detect FRYL in control (transfected with NB1) and experimental lysate (transfected with NB1205).

### Assessment of Effect of NB1 and NB1205 on *in vivo* CRPC Tumor Growth

We evaluated the effect of NB1205 and NB1 on *in vivo* tumor growth. All animal studies were approved by the Institutional Animal Care and Use Committee at Weill Cornell Medicine and were performed in accordance with relevant guidelines and regulations. Male NOD/SCID gamma NSG) mice were implanted subcutaneously into the right flank with 1 × 10^6^ C4-2B CRPC cells. Tumors were allowed to grow and mice were randomized into two groups of three mice per group on day 61. Both groups were administered with daily subcutaneous injections of 100 μl of either 100 nM of control scramble duplex (NB1) or miR-1205 duplex (NB1205) for 10 days. Tumor volume (length, width, height) was determined using a vernier caliper and a scale was used to measure mouse body weight daily. All tumor-bearing mice were euthanized at day 70.

### Annexin V/Propidium Iodide Staining

Annexin V-FITC and propidium iodide staining assay was performed as previously described. LNCaP and C4-2B cells were transfected with either the control scramble oligonucleotide or miR-1205 mimic and after 48 h the cells were harvested, stained and examined through flow cytometry.

### Statistical Analysis

Data was collected from multiple independent experiments. All results are presented as mean ± standard error of the mean (SEM). Unless otherwise indicated, analysis of statistical significance of differences between groups was performed using two-tailed Student’s *t*-test, and only values with *p* < 0.05 were deemed significant. Data presented were analyzed using one-way ANOVA test, multiple comparison Tukey *post hoc* test, and/or independent Student’s *t*-test as described (Das et al., 2016a).

**FIGURE 1:**
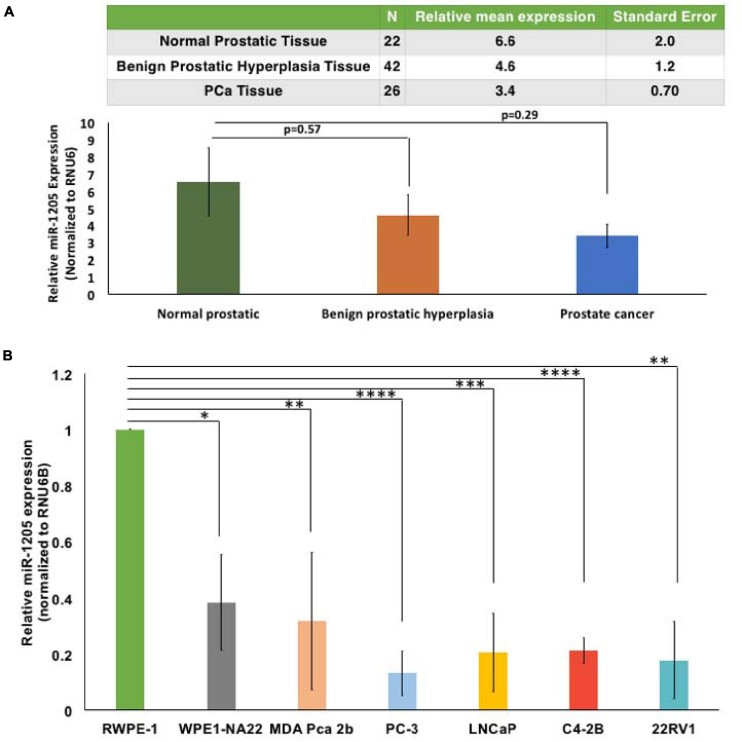
MicroRNA-1205 is underexpressed in PCa tissues and cells. **(A)** RNA expression was determined by RT-qPCR from a cohort of histologically confirmed normal prostatic (*n* = 22), benign prostatic hyperplasia (*n* = 42), and PCa (*n* = 26) histologically confirmed tissues obtained from prostatectomy or transrectal ultrasound-guided biopsies. Tissues were collected in compliance with Institutional Ethics Board approved protocol. One-way ANOVA analysis determined changes in the relative expression of miR-1205 between groups [*F*(2, 87) = 1.153] and a Tukey *post hoc* test revealed decreased miR-1205 expression in benign (4.61 ± 7.5) and malignant tumors (3.39 ± 3.53) when compared to normal tissues (6.55 ± 9.5). **(B)** RWPE-1, WPE1-NA22, MDA-PCa-2b, PC-3, LNCaP, C4-2B, and 22RV1 cells were used to study the expression of miR-1205 in PCa. RT-qPCR analysis showed a significant decrease of miR-1205 RNA expression in PCa cells when compared to normal epithelial RWPE-1 cells. ^∗^*p* < 0.05, ^∗∗^*p* < 0.01, ^∗∗∗^*p* < 0.001, and ^****^p < 0.0001 compared to the control. Data is presented as mean and bars represent mean ± SD (*n* = 3).

**FIGURE 2:**
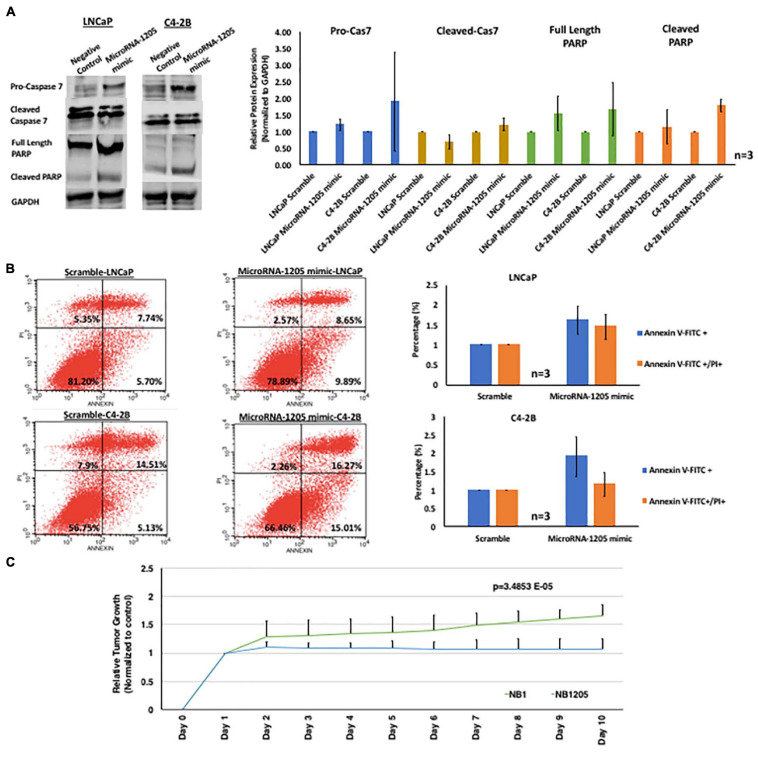
miR-1205 exerts a tumor suppressive effect *in vitro* and *in vivo*. **(A)** LNCaP and C4-2B cells transfected with microRNA-1205 mimic lead to an increased activation of the executioner caspase 7 and PARP cleavage when compared to cells transfected with NB1. Histogram represents quantification of caspase 7, cleaved caspase 7, full length PARP and cleaved PARP normalized to GAPDH. Data is represented as mean ± SD (*n* = 3). **(B)** The effect of miR-1205 overexpression on apoptosis was further assessed with AnnexinV/PI staining in LNCaP and C4-2B cells. Overexpression of miR-1205 led to an increase in apoptosis in LNCaP and C4-2B cells when compared to cells transfected with a negative control scramble. Histogram represents percentage of cells gated for Annexin V-FITC or Annexin V-FITC/PI and normalized to scramble. Data is represented as mean ± SD (*n* = 3). **(C)** Male NOD/SCID gamma mice were subcutaneously implanted with C42B CRPC cells. Mice were randomized into two groups of 3 mice each and administered NB1 or NB1205 for 10 days (dosage of 100 mM). Effects were assessed by daily measurements of tumor volume. Inhibition of tumor growth was observed in mice treated with NB1205. Data is presented as average of tumor volume. There was a statistically significant difference between NB1 and NB1205 groups from days 1 to 10 as determined by one-way ANOVA [*F*(1, 18) = 4.414, *p* = 3.4853E-05].

**FIGURE 3:**
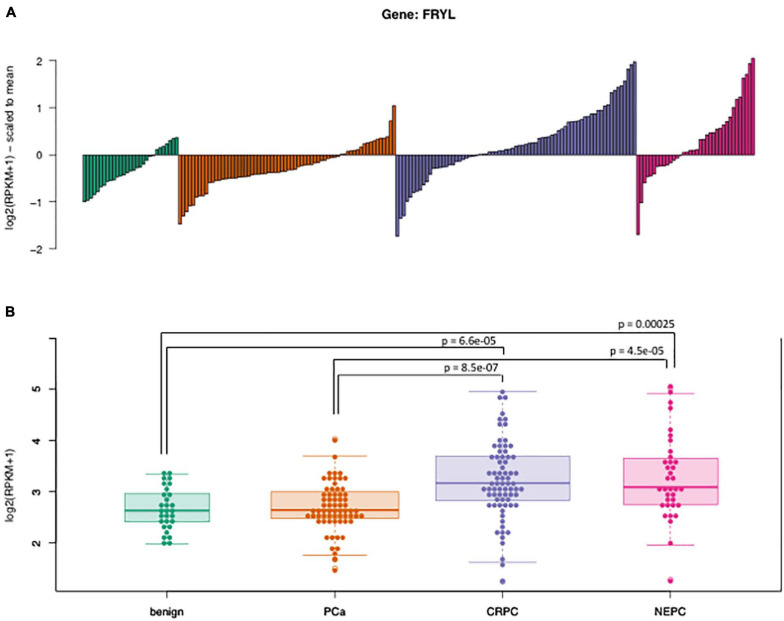
Expression of FRYL in the WCM cohort. **(A)** The waterfall plot shows the median-scaled values of FRYL FPKMs across the cohort for each sub-group (benign, PCa, CRPC, and NEPC) and **(B)** the boxplots illustrate the absolute distribution of the log-2 transformed expression levels. The expression of FRYL is significantly upregulated in advanced prostate cancers (CRPC, NEPC) compared to either benign prostate or localized PCa tumors. Pairwise comparisons (Wilxocon test) were performed to determine significance (*p*-value).

**FIGURE 4:**
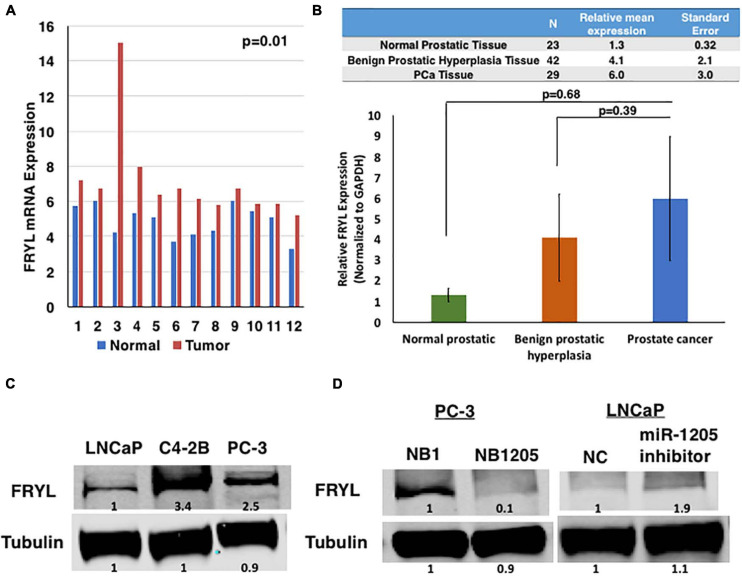
FRYL is overexpressed in PCa and is regulated by miR-1205. **(A)** FRYL was significantly overexpressed in PCa tissues when compared to normal tissues {ANOVA [*F*(1, 22) = 4.3, *p* = 0.009]. **(B)** Using thesame cohort of tissues in Figure 1A, FRYL overexpression was observed in PCa tissues when compared to normal prostatic tissues. **(C)** Western blot analysis of FRYL in CRPC C4-2B, small cell prostate carcinoma PC-3 cells and androgen-sensitive LNCaP cells. **(D)** FRYL protein levels in PC-3 cells after inhibition of miR-1205 with NB1205. Data is presented as ± SD.

**FIGURE 5:**
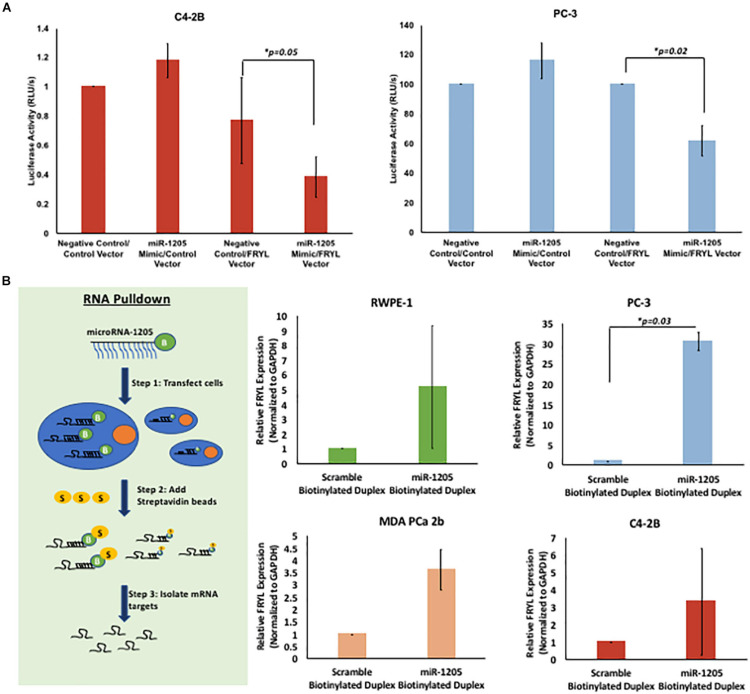
miR-1205 directly binds to the 3′UTR of FRYL. C4-2B and PC-3 cells were co-transfected with Genecopoeia pEZX-MT06 miRNA reporter empty vector or FRYL plasmid with a non-targeting negative control or miR-1205 mimic for 24 h. The Fluc and Rluc activity was measured. Luciferase activity is normalized to the negative control luciferase activity. *P < 0.05 compared to Negative control/FRYL Vector (*n* = 3). **(A)** C4-2B cells transfected with miR-1205 and the 3′UTR of FRYL in a luciferase expressing vector revealed a significant decrease in luciferase activity when compared to control cells, indicating direct binding of miR-1205 to the 3′UTR of FRYL. **(B)** Overview of RNA pull down assay is demonstrated. Overall enrichment of FRYL was observed in RWPE-1, PC-3, MDA PCa 2b and C4-2B cells transfected with the biotinylated miR-1205 duplex (NB1205). *p < 0.05 compared to Scramble Biotinylated Duplex (*n* = 3). Data is presented as mean and bars represent standard error of the mean ± SD.

## Results

### MicroRNA-1205 Is Underexpressed in PCa Tissue and Cells

To determine the role of miR-1205 in PCa, a cohort of histologically confirmed normal prostatic (*n* = 22), benign prostatic hyperplasia (*n* = 42), and PCa (*n* = 26) tissues were obtained from prostatectomy or transrectal ultrasound-guided biopsies and the levels of miR-1205 RNA expression were examined. One-way ANOVA analysis determined changes in the relative expression of miR-1205 between groups [*F*(2, 87) = 1.153]. A Tukey *post hoc* test revealed that miR-1205 expression was twofold lower in benign tissue (4.61 ± 7.5), and about threefold lower in PCa tissue (3.39 ± 3.53), when compared to normal prostatic tissue (6.55 ± 9.5) (Figure 1A). This data demonstrates that miR-1205 is underexpressed in human prostatic tumor tissue suggesting that loss of miR-1205 function may drive progression of solid prostatic tumors. To further verify the underexpression of miR-1205 in PCa, a panel of human PCa cell lines including RWPE-1 (normal prostate epithelial cells) (Bello et al., 1997), WPE1-NA22 (RWPE-1 cells transformed with MNU; non-invasive) (Webber et al., 2001), MDA-PCa-2b (PCa adenocarcinoma) (Navone et al., 1997), PC-3 (small cell prostatic carcinoma) (Kaighn et al., 1979; Tai et al., 2011), LNCaP (androgen-sensitive PCa adenocarcinoma) (Horoszewicz et al., 1980), C4-2B (androgen-insensitive PCa adenocarcinoma) (Thalmann et al., 2000), and 22RV1 (androgen-insensitive PCa adenocarcinoma) (Sramkoski et al., 1999; Dehm et al., 2008) were used to asses mRNA expression. An overall significant decrease of miR-1205 expression was observed in PCa cells when compared to normal epithelial RWPE-1 cells (Figure 1B). Interestingly, androgen-sensitive cells (WPE1-NA22, MDA PCa 2b, and LNCaP) displayed ∼60% decrease in miR-1205 expression, whereas androgen-insensitive (PC-3, C4-2B and 22RV1) cells displayed ∼80% reduction of miR-1205 expression in comparison to RWPE-1 cells. These observations suggest that miR-1205 may influence the phenotype and progression of aggressive PCa. Furthermore, underexpression of miR-1205 could indicate that it may have tumor suppressive functions that could drive PCa aggressiveness.

### MicroRNA-1205 Synthetic Analog, NB1205, Suppresses Tumor Growth in Xenograft CRPC Mouse Model

To assess whether miR-1205 is involved in tumor suppression in PCa, we investigated the function of miR-1205 *in vitro* by examining its effect on proliferation and apoptosis of PCa cells. We designed a synthetic biotinylated miR-1205 duplex (NB1205, patent pending) and a control synthetic biotinylated scramble duplex (NB1) to be used as tools for studying the function of miR-1205. MTT assays revealed that C4-2B cells transfected with NB1205 did not significantly affect cell proliferation when compared to cells transfected with NB1, suggesting that miR-1205 is not involved in the proliferation PCa (Figure 2A). However, when examining apoptotic markers we observed an increase in cleaved caspase 7 and cleaved PARP in cells overexpressing miR-1205, indicating that exogenous delivery of miR-1205 induces DNA damage and activates the apoptotic pathway (Figure 2B). A modest increase of pro-caspase 3 and cleaved caspase 9 was also observed in C4-2B cells transfected with a miR-1205 mimic (Supplementary Figure 2B). To further support the role of miR-1205 in apoptosis we stained LNCaP and C4-2B cells transfected with a miR-1205 mimic and control scramble oligonucleotide with Annexin V-FITC and propidium iodide. We observed an increase of apoptosis occurrence in LNCaP and C4-2B cells overexpressing miR-1205 when compared to cells transfected with a negative control scramble oligonucleotide (Figure 2B). We next performed an *in vivo* study to determine whether miR-1205 can suppress tumorigenesis in PCa. Mice treated with NB1205 displayed significantly smaller tumor volumes {one-way ANOVA [*F*(1, 18) = 4.414, *p* = 3.4853E-0]}, when compared to mice intervened with NB1 (Figure 2C). These results, taken together, strongly suggest that miR-1205 have tumor suppressive properties in aggressive PCa.

### FRYL Is Identified as a Downstream Molecular Target of miR-1205

To further investigate the tumor suppressive effects of miR-1205 in PCa, putative targets of miR-1205 were determined using miRNA molecular target predication algorithms (such as miRBase and miRDB). Each target was screened using the galaxy web platform (see text footnote 1) for differential expression patterns in PCa. Consequently, Fry-like (FRYL) was identified as a putative target of miR-1205. Very little is known about FRYL, however, the c-terminus of FRYL is often observed to be fused to the mixed lineage leukemia (MLL) gene associated with treatment related acute lymphoblastic leukemia (ALL) (Hayette et al., 2005; Sait et al., 2007). FRYL has almost identical functional domains to the well characterized Fry protein, which functions as protein involved in many processes including cell polarization during morphogenesis, dendritic branching and tiling, spindle organization during division and gene expression (Nagai and Mizuno, 2014). However, the function of FRYL as a target of miR-1205 has never been characterized, and FRYL’s relevance in PCa remains unknown.

To examine the differential expression patterns of FRYL in PCa, we first evaluated the mRNA expression of FRYL by RNAseq in a subset of prostate cancers (Figure 3). FRYL expression is significantly higher in CRPC and NEPC patient tumor samples when compared to prostate benign tumors (Wilcoxon test; benign vs. CRPC *p* = 6.6e-05, benign vs. NEPC *p* = 0.00025, PCa vs. CRPC *p* = 8.5e-07, and PCa vs. NEPC p = 4.5e-05). Additionally, we performed whole transcriptome analysis on prostatic and adjacent normal tissue obtained from fourteen PCa patients and observed FRYL overexpression was observed in PCa tissue when compared to normal tissue (Figure 4A; Ren et al., 2012). Moreover, FRYL overexpression was observed in PCa tissues when compared to normal prostatic tissues (using the same cohort of patients from Figure 1) (Figure 4B). Altogether, the FRYL expression among all three cohorts demonstrates for the first time that FRYL is overexpressed in PCa, CRPC and NEPC tissue. Lastly, we observed an overexpression of FRYL at mRNA and protein levels in CRPC C4-2B and small cell prostate adenocarcinoma PC-3 cells when compared to androgen-sensitive LNCaP cells, indicating a putative role of FRYL in aggressive PCa (Figure 4C and Supplementary Figure 2).

To identify miR-1205 targeting of FRYL, PC-3 cells and LNCaP cells were transfected with a synthetic analog of miR-1205, NB1205, and a miR-1205 inhibitor, respectively (Figure 4D and Supplementary Figure 2). We observed a downregulation of FRYL in response to exogenous NB1205 transfected into PC-3 cells which was not observed after treatments with the synthetic scramble analog, NB1 in PC-3 cells. To determine whether miR-1205 regulates FRYL, LNCaP cells were transfected with a miR-1205 inhibitor and FRYL expression was studied. Interestingly, data showed that FRYL levels increased after inhibition of miR-1205. Moreover, miR-1205 underexpression moderately correlates with FRYL overexpression in PCa tissue (Supplementary Figure 1). Altogether, these results suggest that miR-1205 may be regulating FRYL by targeting FRYL mRNA, leading to its degradation.

### FRYL Is a Direct Molecular Target of miR-1205

To validate FRYL as a downstream molecular target of miR-1205, a Luc-Pair Duo-Luciferase Assay was performed to determine whether miR-1205 binds to the 3′UTR of FRYL. The 3′UTR of FRYL contains a total of three putative binding sites to the seed region of miR-1205 (Supplementary Figure 4A). C4-2B, PC-3 and LNCaP cells were either transfected with Genecopoeia pEZX-MT06 miRNA reporter containing the 3′UTR of FRYL or an empty vector. Additionally, cells were co-transfected with a non-targeting scramble negative control, or with miR-1205 mimic. A significant decrease in luciferase activity was observed in cells transfected with miR-1205 mimic and the miRNA luciferase reporter construct containing the 3′UTR of FRYL, indicating direct binding of miR-1205 to FRYL (Figure 5A and Supplementary Figure 4B).

To further examine this observation, an RNA pulldown assay was performed to assess the binding of miR-1205 to FRYL mRNA. RWPE-1, PC-3 and MDA PCa 2b cells were transfected with either a biotinylated miR-1205 duplex or scramble biotinylated duplex for 24 h. We observed enrichment of FRYL in all four cell lines that were transfected with biotinylated miR-1205, further indicating that miR-1205 directly binds to FRYL mRNA in PCa cells (Figure 5B and Supplementary Figure 3).

### miR-1205 Regulation of FRYL mRNA May Play a Role in PCa NED Development

**FIGURE 6:**
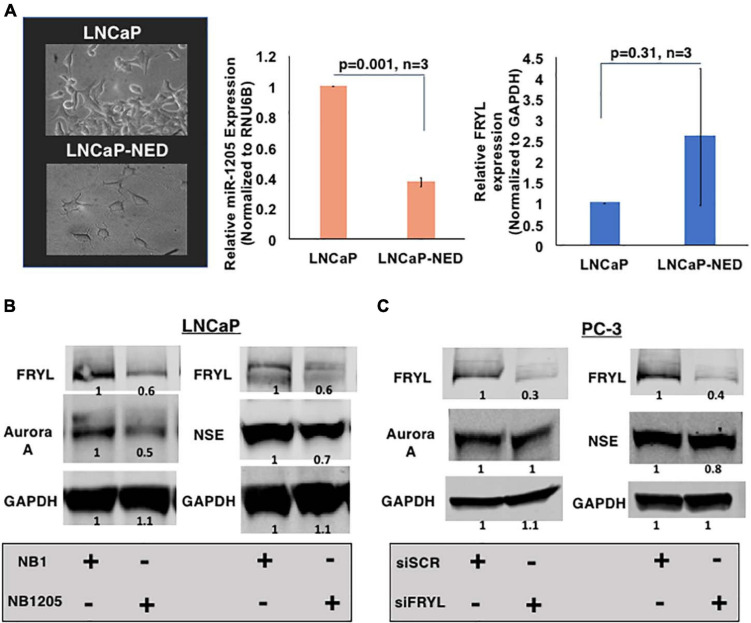
miR-1205 regulation of FRYL mRNA may play a role in PCa NED development. **(A)** Morphology (left) and FRYL mRNA levels (right) of LNCaP cells after inducing NED by culturing androgen-sensitive LNCaP cells under androgen deprivation conditions. **(B)** A negative control scramble oligonuceltide (NB1) and miR-1205 mimic (NB1205) were transfected in LNCaP cells to assess induction of NED in androgen-sensitive cells. MiR-1205 overexpression led to a decrease in neuroendocrine markers, NSE and Aurora A protein expression **(C)** NSE and Aurora A levels after siRNA-mediated silencing of FRYL in PC-3 cells. Data is presented as mean and bars represent mean ± SD.

As mentioned previously, FRYL is predicted to regulate dendritic branching leading to our hypothesis that FRYL plays a role in the progression of PCa NED, a resulting mechanism due to ADT resistance. The morphology of neuroendocrine cells is very distinct, in which dendrite-like protrusions are observed (di Sant Agnese and de Mesy Jensen, 1987; Yuan et al., 2007). To test our hypothesis, we induced NED *in vitro* and assessed the expression levels of miR-1205 and FRYL. NED was induced by culturing LNCaP cells in RPMI-1640 medium supplemented with 10% charcoal-stripped FBS (androgen deprivation conditions). Cells were maintained in these conditions for fourteen days and expression levels of miR-1205, FRYL and NED markers (chromogranin A and NSE) along with morphological changes were observed via RT-qPCR, western blotting and light microscopy (Figure 6A and Supplementary Figure 5A). After LNCaP-NED was induced, we observed an overexpression of FRYL mRNA whereas miR-1205 was significantly underexpressed when compared to undifferentiated LNCaP cells, indicating a putative role of miR-1205 regulation of FRYL in PCa NED.

To determine whether the miR-1205/FRYL regulatory pathway is important in PCa NED, miR-1205 was overexpressed in LNCaP cells and neuroendocrine marker expression was assessed. When NB1205 (miR-1205 mimic) was transfected into LNCaP cells, we observed a decrease in expression of neuroendocrine markers NSE and Aurora A when compared to LNCaP cells transfected with NB1 (scramble oligonucleotide) (Figure 6B and Supplementary Figure 5B). This observation suggests that miR-1205 may be involved in NED. We additionally observed induction of NED by inhibiting miR-1205 in LNCaP cells (Supplementary Figure 5D). To determine whether miR-1205 regulation of FRYL is involved in NED, FRYL was silenced in PC-3 small cell prostatic carcinoma cells. Interestingly, we observed no changes in NSE expression and only a 20% decrease in aurora A expression, suggesting that miR-1205 regulation of FRYL may not significantly regulate NED in PCa cells (Figure 6C and Supplementary Figure 5).

## Discussion

This study demonstrates novel findings into the molecular mechanisms of PVT1-encoded miR-1205 in PCa. We discovered that miR-1205 was underexpressed in histologically confirmed PCa tissue when compared to normal prostatic tissue. This underexpression was further observed among a large panel of PCa cell lines when compared to normal prostate epithelial cells. While miR-1205 does not affect cell proliferation of PCa cells, we discovered that overexpression of miR-1205 induces apoptosis and suppresses PCa tumor growth. We further describe the molecular actions of miR-1205 through regulation of its validated target, FRYL. FRYL is overexpressed in PCa tissues and aggressive PCa cell line models. Moreover, miR-1205 expression leads to the FRYL protein inhibition through direct targeting of the 3′UTR of FRYL. Lastly, we discovered that miR-1205 may be involved in neuroendocrine differentiation of androgen-dependent PCa cells, suggesting a role in aggressive PCa.

It was previously reported that miR-1205 is expressed at very low levels among cancer cell lines (not including PCa cell lines), questioning its role in tumorigenesis (Beck-Engeser et al., 2008; Huppi et al., 2008). A recent report by Wang et al. (2019) of the role of the miR-1205-EGLN3 axis in PCa suggests that there is somatic DNA amplification at the locus for PVT1-encoded miRs-1204-1208 and that this is correlated with miR-1205 overexpression in PC-3 cells. In this present study, however, when miR-1205 expression was examined using a panel of PCa cell lines, we discovered that miR-1205 is significantly underexpressed in PCa cell lines, when compared to normal prostate epithelial cells. Additionally, we observed that miR-1205 was underexpressed in PCa tissues, when compared to normal prostatic tissue, suggesting that loss of miR-1205 may be occurring during PCa tumorigenesis. While somatic amplification may occur at the locus for PVT1-encoded microRNAs, regulation at the promoter region was not investigated by Bello et al. (1997). There is evidence that PVT1-encoded microRNAs do not share the same promoters and may be subjected to differential regulation (Cho et al., 2018). A closer look into the regulation at the promoter region of miR-1205 will further elucidate mechanisms of miR-1205 expression and action in PCa cells. Moreover, there is evidence that methyltransferases are important for the methylation of primary miRNA, allowing for recognition and processing by DGCR8 during miRNA biogenesis (Alarcon et al., 2015). Loss of certain methyltransferases can lead to reduced binding of primary miRNAs to DGCR8 leading to cessation of mature miRNA. Further studies into the upstream regulation of PVT1-encoded miRNAs, including miR-1205, could reveal insight on how miRNAs could be underexpressed in cancers. Nevertheless, we demonstrate for the first time that mature miR-1205 transcript are lower in PCa cells and that it’s loss may contribute to cancer progression. There is now an abundance of evidence indicating that miRNAs can function as tumor suppressors and/or oncogenes, ultimately enhancing the progression of tumorigenesis. Furthermore, in-depth investigation on their potential role in cancer diagnosis, prognosis and treatments are ongoing. MiRNAs are small molecules that can be found in biological fluids, such as urine and blood, whereby their detection and quantification of their expression levels can be used as biomarkers to link cancer incidence and progression. Moreover, with a proper delivery system, miRNAs can be used as inhibitors or mimics via anti-miR technology or replacement therapies, respectively, to treat cancers (Stenvang et al., 2012; Hosseinahli et al., 2018). As such, we were interested in discovering the clinical significance of miR-1205 in PCa to subsequently demonstrate its potential use as a novel therapeutic for treating aggressive PCa. In this study, we present the development of a novel tool and demonstrate its use for *in vitro* and *in vivo* techniques. A biotinylated synthetic analog of miR-1205 (NB1205) and a control biotinylated synthetic scramble duplex (NB1) was generated in effort to test our hypothesis that miR-1205 is a tumor suppressor. Using this tool, we first showed that while miR-1205 does not regulate cell proliferation, it does induce apoptosis through caspase and PARP cleavage. Furthermore, we established that NB1205 suppressed tumors in *in vivo* CRPC model. Ultimately, this data suggests that miR-1205 has tumor suppressive functions by inducing apoptosis of aggressive PCa cells.

MiRNAs comprise about 1–5% of the human genome, but regulates about 30% of protein coding genes (Berezikov et al., 2005; MacFarlane and Murphy, 2010). These small non-coding RNAs mediate mRNA gene silencing through RNA-interfering (RNAi) pathways. Partial binding to the 3′UTR of an mRNA results in translational inhibition, whereas extensive binding to a target mRNA results in degradation. Moreover, each miRNA can have an average of 200 mRNA targets, illustrating their importance in regulating multiple cellular processes (Krek et al., 2005). We were interested in the mechanisms involved in the tumor suppressor role of miR-1205 and therefore we examined the putative targets of miR-1205 where FRYL was identified and validated as a target of miR-1205. FRYL was first identified as a novel fusion partner of the mixed lineage leukemia (*MLL*) gene that was reported in patients who developed treatment-related acute lymphoblastic leukemia (ALL) (Hayette et al., 2005; Sait et al., 2007). The *MLL* gene has over 35 partner genes, which produce chimeric proteins containing the N-terminus of *MLL* and C-terminus of its fusion partner gene, such as *FRYL*. We established for the first time the overexpression FRYL mRNA and protein in PCa, CRPC, and NEPC cell lines and tissues, further implicating FRYL as a putative oncogenic factor in cancers such as PCa and ALL. Investigation of the MLL and FRYL fusion protein led to the discovery that the c-terminus of FRYL exhibits transcriptional activation properties, indicating that FRYL is a transcriptional activator (Hayette et al., 2005). Therefore, it will be interesting to further investigate the role of FRYL as a transcription factor and its possibility to act as a fusion partner for genes involved in cancer progression.

Amplification and overexpression of *MYCN* and *AURKA* genes have been observed in confirmed t-NEPC tissue, and both induce a neuroendocrine cell phenotype in prostatic adenocarcinoma cells through transcriptional reprogramming mechanisms (Mosquera et al., 2013; Dardenne et al., 2016). This mechanism, described as neuroendocrine differentiation (NED) is thought to occur as a means for survival against ADTs due to the lack of AR expression and dependence on AR signaling for survival among neuroendocrine cells. In addition to NED, it is hypothesized that NEPC cell population can also increase due to PCa stem cells that are increasingly differentiating into neuroendocrine cells. It is not clear whether NEPC cells increase via cancer stem cells and/or transdifferentiation in aggressive disease. This field requires further understanding as more men are diagnosed with PCa and therefore, develop resistant and untreatable cancers. Whether the origins of neuroendocrine cells within a PCa tumor emerge from PCa stem cells or through cellular reprogramming mechanisms, novel findings into the mechanisms that drive NEPC will not only help create new therapies for treatment, but also to shed some light on the role of neuroendocrine cells within the prostate.

The human genome contains two *fry*-related genes including *fry* and *FRYL*, which share almost identical amino acid compositions among conserved domains (Nagai and Mizuno, 2014). Fry has been studied in multiple models such as, *Drosophila melanogaster*, and plays a crucial role in multiple cellular processes including, dendritic branching during development, morphogenesis, cell division and cell polarization (Emoto et al., 2004). Moreover, there is evidence that aurora A binds to the c-terminal region of the mammalian fry protein and induces phosphorylation to promote proper spindle formation during mitosis (Ikeda et al., 2012). However, it is unknown whether the FRYL protein shares these functions. Neuroendocrine cells located within the prostate epithelium have features of dendrite-like morphologies, which led us to investigate the role of FRYL in NED induced by ADT (di Sant Agnese, 1992; Yuan et al., 2007). By culturing LNCaP androgen-sensitive cells under androgen deprivation conditions, we observed a significant decrease in miR-1205 expression and an increase in FRYL RNA expression. Additionally, we observed that FRYL mRNA expression was significantly higher in CRPC and NEPC patient tumor samples, when compared to prostate benign tumors. These results led us to hypothesize that miR-1205 regulation of FRYL is involved in NED in aggressive PCa. Our data showed that overexpression of miR-1205 in androgen sensitive cells increased the expression levels of neuroendocrine markers (NSE and aurora A) and FRYL suggesting that miR-1205 regulation of FRYL may be involved in NED. However, when we investigated whether NED occurs directly through FRYL protein signaling, loss of FRYL did not induce changes in NSE expression and only induced a 20% decrease in aurora A expression, indicating that miR-1205 may not be regulating NED through FRYL regulation. However, further investigation is required. Fry and FRYL contain very similar structures within their c-terminal domains, which contains transcriptional activation properties. While loss of FRYL may not significantly affect the transcriptional regulation of aurora A, loss of aurora A could interfere with post-translational modifications within the c-terminal domain of FRYL that may overall contribute to regulation of NED. Therefore, further investigation into the phosphorylation profile of FRYL is needed. Nevertheless, our data ultimately suggests that miR-1205 regulates NED and therefore, further investigation into miR-1205 targets may uncover novel mechanisms that drive NED.

## Conclusion

In conclusion, we reveal for the first time that PVT1-encoded miR-1205 is underexpressed in PCa. Our data suggests that miR-1205 may have tumor suppressive functions through the regulation of FRYL in CRPC PCa. However, loss of miR-1205 may induce NED, a phenotype that appears in aggressive PCa, but may act independently of FRYL. Further investigation of miR-1205 in this mechanism may reveal novel insights on the progression of PCa.

## Data Availability Statement

The datasets presented in this study can be found in online repositories. The names of the repository/repositories and accession number(s) can be found below: NCBI’s GEO under accession numbers GSE117306, GSE117430, GSE117281, and GSE117282.

## Ethics Statement

The studies involving human participants were reviewed and approved by the City University of New York. The patients/participants provided their written informed consent to participate in this study. The animal study was reviewed and approved by Weill Cornell Medicine.

## Author Contributions

MN performed all experiments and wrote manuscript and prepared figures. FL and TG assisted with *in vivo* work in Figure 2C. ATO and EO-O recruited and managed patients, provided the human tissue samples and helped prepare the corresponding method section for data in Figure 1A. TA and KK performed transcriptome analysis in Figure 4A. CP assisted with statistical analysis in Supplementary Figure 1. PD and AS analyzed FRYL in normal prostate vs localized PCa vs CRPC vs NEPC and performed statistical analysis in Figure 3. OOO supervised all the work from conception to manuscript preparation and writing of final version of the manuscript. MN and OOO revised final version of the manuscript.

## Conflict of Interest

OOO was Co-Founder of NucleoBio, Inc., a City University of New York start-up biotechnology company. The remaining authors declare that the research was conducted in the absence of any commercial or financial relationships that could be construed as a potential conflict of interest.

## Publisher’s Note

All claims expressed in this article are solely those of the authors and do not necessarily represent those of their affiliated organizations, or those of the publisher, the editors and the reviewers. Any product that may be evaluated in this article, or claim that may be made by its manufacturer, is not guaranteed or endorsed by the publisher.

## Footnotes

1
https://usegalaxy.org

2
http://hgdownload.soe.ucsc.edu/goldenPath/hg19/bigZips
