# Supplementary material for: MicroRNA-1205 Regulation of FRYL in Prostate Cancer
Source: Front Cell Dev Biol. 2021 Jul 27;9:647485. doi: 10.3389/fcell.2021.647485 (PMC8354587; doi:10.3389/fcell.2021.647485)
Supplement: Supplementary file 1 [file Data_Sheet_1.PDF]

Supplementary Material

1.1 Supplementary Figures

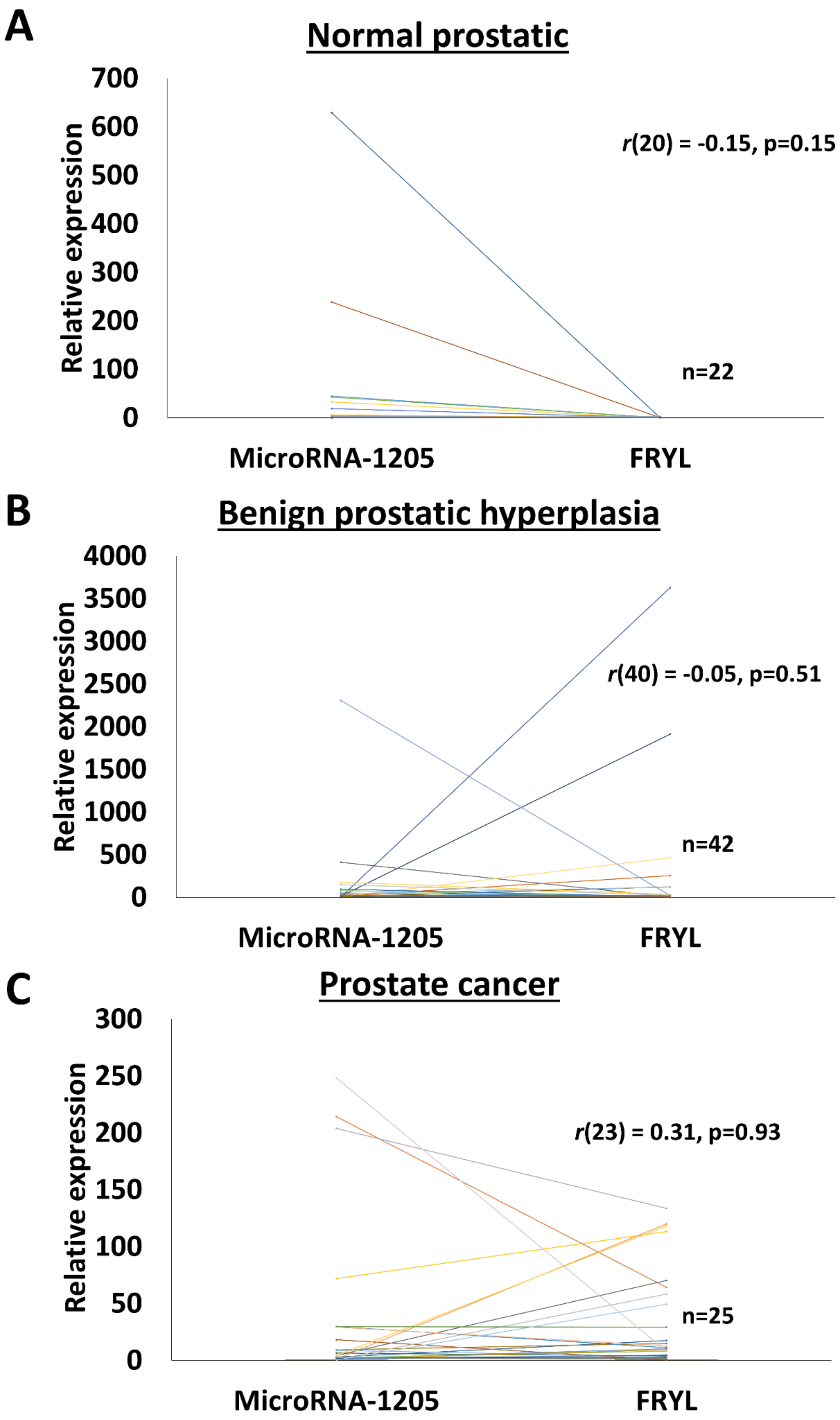
A
Normal prostatic
Relative expression
700
600
500
400
300
200
100
0
r(20) = -0.15, p=0.15
n=22
MicroRNA-1205
FRYL
B
Benign prostatic hyperplasia
Relative expression
4000
3500
3000
2500
2000
1500
1000
500
0
r(40) = -0.05, p=0.51
n=42
MicroRNA-1205
FRYL
C
Prostate cancer
Relative expression
300
250
200
150
100
50
0
r(23) = 0.31, p=0.93
n=25
MicroRNA-1205
FRYL

Supplementary Figure 1. MicroRNA-1205 expression was determined by RT-qPCR from a cohort of histologically confirmed (A) normal prostatic (n=22), (B) benign prostatic hyperplasia (n=42), and (C) PCa (n=25) histologically confirmed tissues obtained from prostatectomy or transrectal ultrasound-guided biopsies. Tissues were collected in compliance with Institutional Ethics Board approved protocol. FRYL and miR-1205 expression were compared in each data set as represented in the dot plots and Pearson Correlation Coefficient analysis and student's t-test was performed to determine correlation and significance. Expression of miR-1205 and FRYL in PCa tissues were moderately positively correlated ($r(23) = 0.31$, p=0.93), while the correlation between FRYL and miR-1205 in normal and benign tissues were weakly negatively correlated (normal: ($r(20) = -0.15$, p=0.15; benign: ($r(40) = -0.05$, p=51).

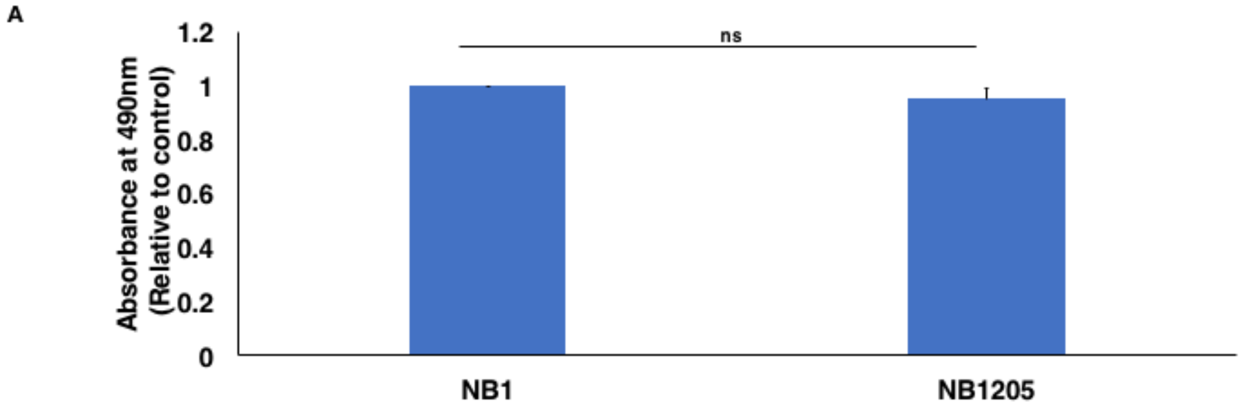

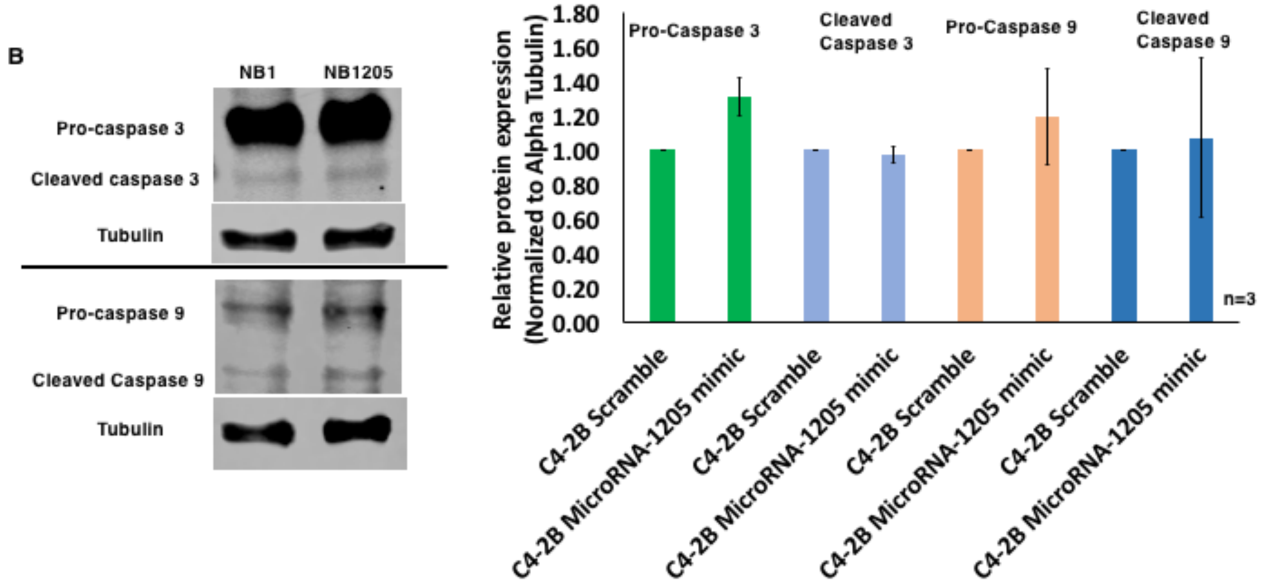

Supplementary Figure 2. (a) MTT assay revealed no significant changes in proliferation when a synthetic analog of miR-1205 (NB1205) was introduced in C4-2B cells compared to the control scramble oligonucleotide (NB1). (b) C4-2B cells were transfected with either a scramble negative control or miR-1205 mimic duplex. Anti-Caspase 3 was first probed and detected at ~35kDa and its cleaved fragment at ~19kDa. The blot was stripped and re-probed with anti-Alpha Tubulin at ~55 kDa as a loading control. Anti-Caspase9 was probed and detected at ~47 kDa and its cleaved fragment at ~37-35kDa. The blot was also probed with Alpha Tubulin detected at ~55kDa as a

loading control. All quantifications were performed using ImageJ analysis normalized to NB1. The histogram represents the protein/Alpha tubulin ratios normalized to the scramble negative control and is represented as mean +/- SD.

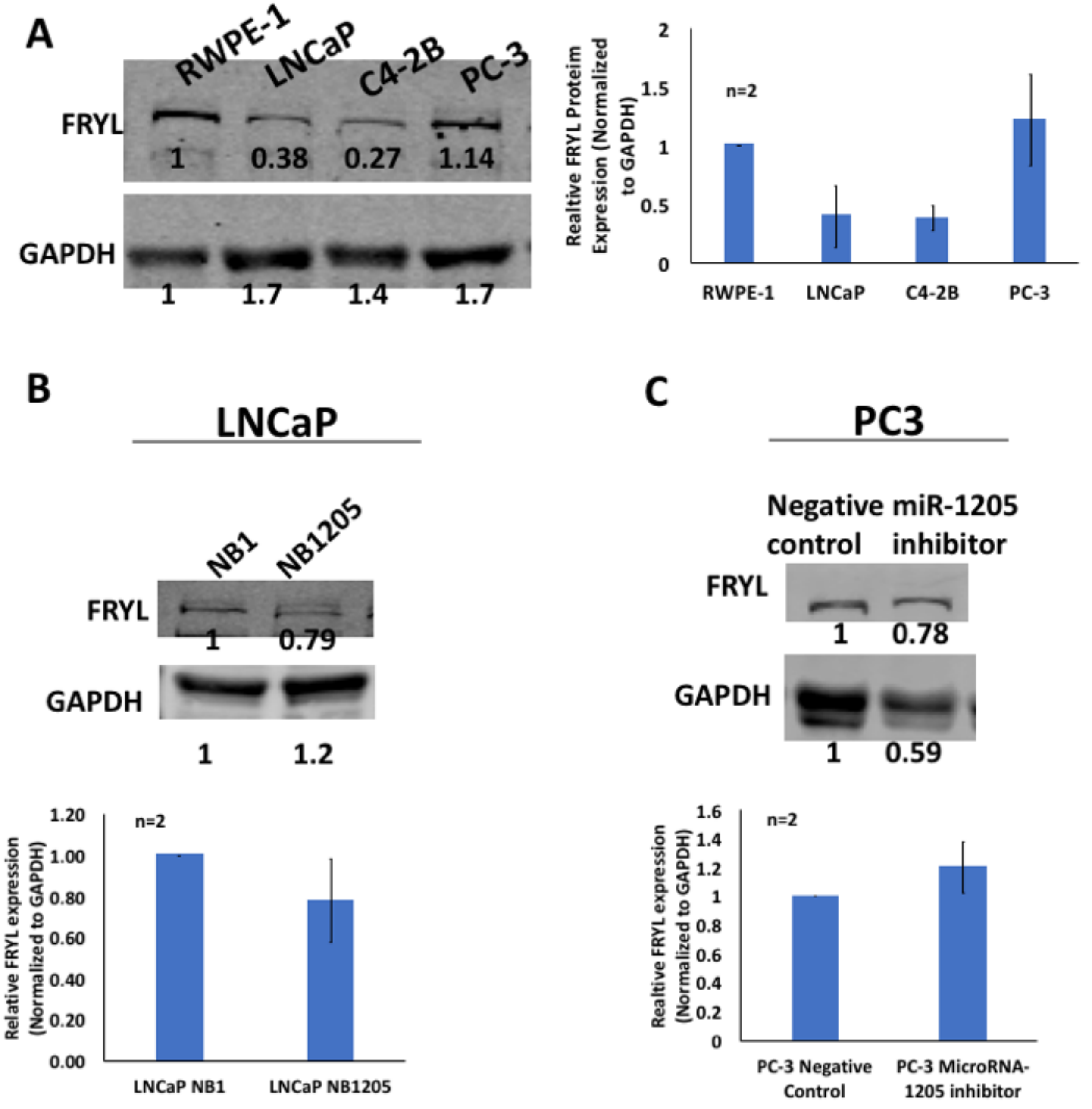

Supplementary Figure 3. FRYL protein expression was assessed in RWPE-1 (normal prostate epithelial cells), LNCaP, C4-2B and PC-3 cells. Anti-FRYL was probed and detected at ~340 kDa via western blotting. Anti-GAPDH was probed as a loading control and detected at ~36kDa. Bands were normalized to RWPE-1. The histogram represents FRYL/GAPDH ratios normalized to RWPE-1 (n=2). b) LNCaP cells transfected with NB1 and NB1205 expression were analyzed for FRYL

protein expression via western blotting. Anti-FRYL was probed and detected at ~340 kDa in LNCaP cells transfected with NB1 and NB1205. Anti-GAPDH was probed as a loading control and detected at ~36kDa. FRYL and GAPDH were normalized to NB1. The histogram represents FRYL/GAPDH ratios normalized to NB1 (n=2). c) PC-3 cells transfected with a negative control oligonucleotide (NC) and miR-1205 inhibitor were analyzed for FRYL protein expression via western blotting. Anti-FRYL was probed and detected at ~340 kDa in PC-3 cells transfected with NCand miR-1205 inhibitor. Anti-GAPDH was probed as a loading control and detected at ~36kDa. FRYL and GAPDH were normalized to NC. The histogram represents FRYL/GAPDH ratios normalized to NC (n=2). All quantifications were performed using ImageJ analysis. Data is represented as mean +/- SD.

A

MicroRNA-1205 binding targets in 3'UTR of FRYL	
Position 890-897 of FRYL 3' UTR	5' ...UAACCAAAGUAGAUGCCUGCAGA...
hsa-miR-1205	3' GAGUUUCGUUUG-GGACGUCU
Position 1117-1123 of FRYL 3' UTR	5' ...UGCAGGCUUUGAAGUCUGCAGAA...
hsa-miR-1205	3' GAGUUUCGUUUGGGACGUCU
Position 2052-2058 of FRYL 3' UTR	5' ...AUAAAAAUGAAUAUACCUGCAGC...
hsa-miR-1205	3' GAGUUUCGUUUGGGACGUCU

B

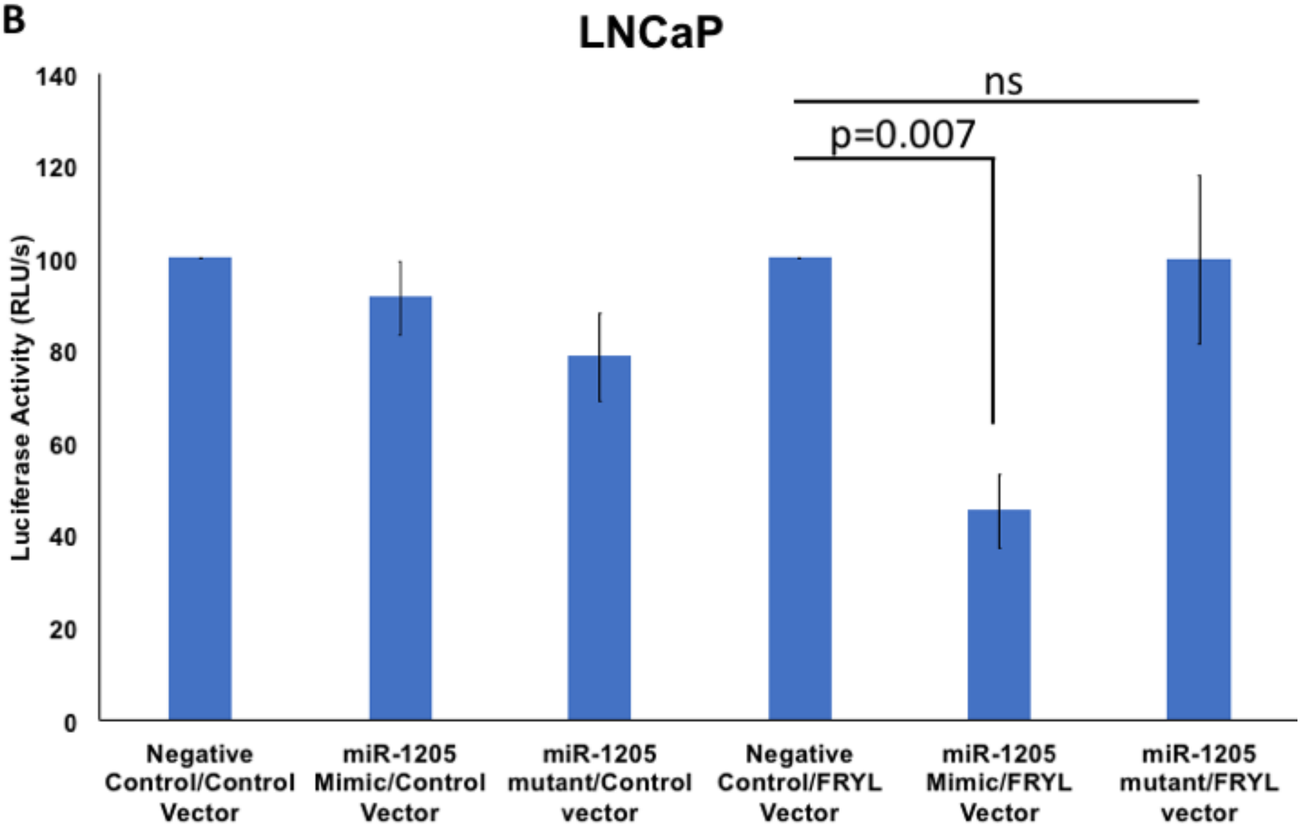

C

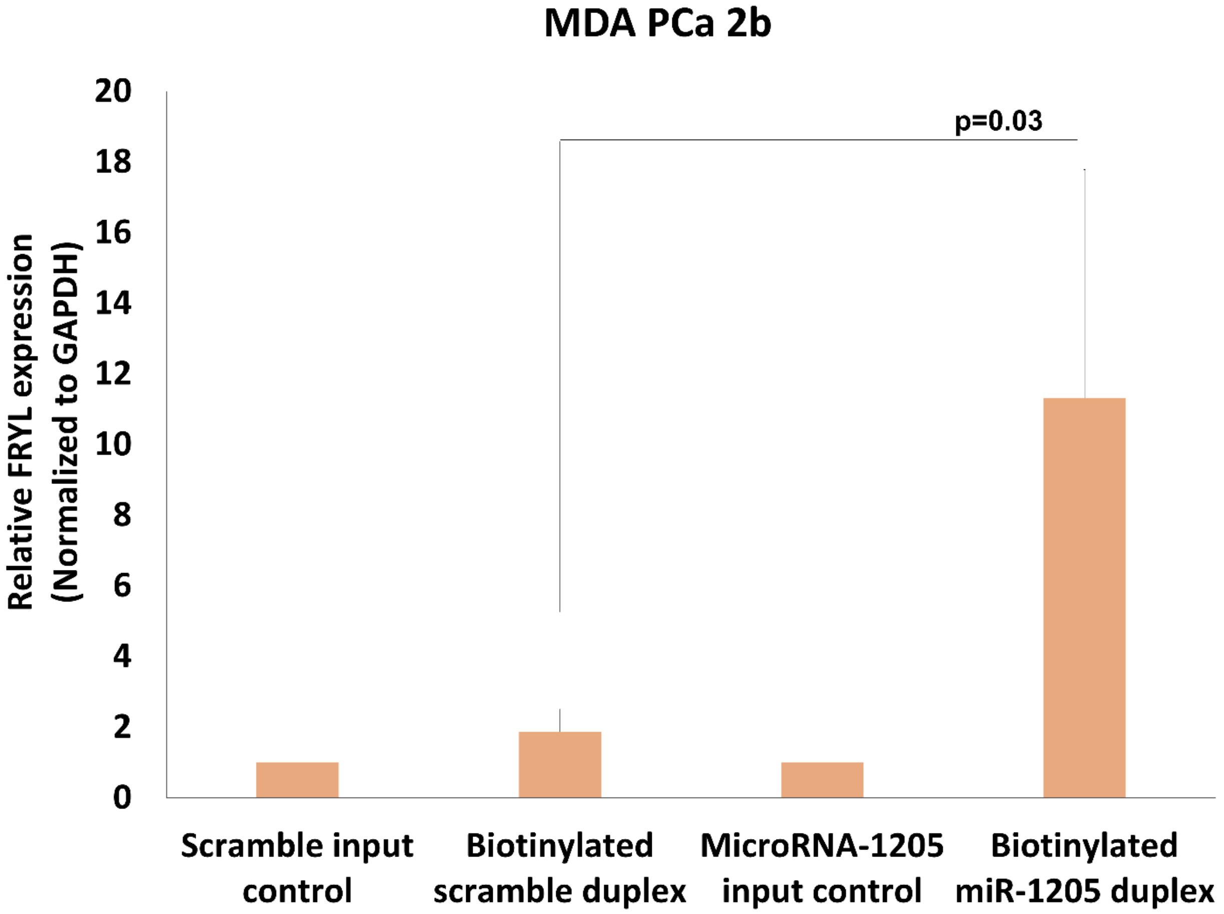

Supplementary Figure 4. MiR-1205 directly binds to the 3'UTR of FRYL. (a) Representation of the three miR-1205 binding sites to FRYL 3-UTR. Matched were taken from TargetScanHuman. (b) LNCaP cells were co-transfected with Genecopoeia pEZX-MT06 miRNA reporter empty vector or FRYL plasmid with a non-targeting negative control, miR-1205 mimic or miR-1205 mutant for 24 hours. The Fluc and Rluc activity was measured. Luciferase activity is normalized to the negative control luciferase activity. *P<0.05 compared to Negative control/FRYL Vector. Data is represented as mean +/- SD. (c) A RNA pull down assay was performed to determine miR-1205 direct binding to FRYL mRNA. Significant enrichment of FRYL was observed in MDA PCa 2b cells transfected with the biotinylayed miR-1205 duplex (NB1205) when compared to cells transfected with the biotinylated scramble duplex (NB1). Data is presented as mean and bars represent standard error of the mean (SEM). *P<0.05 compared to biotinylated scramble duplex.

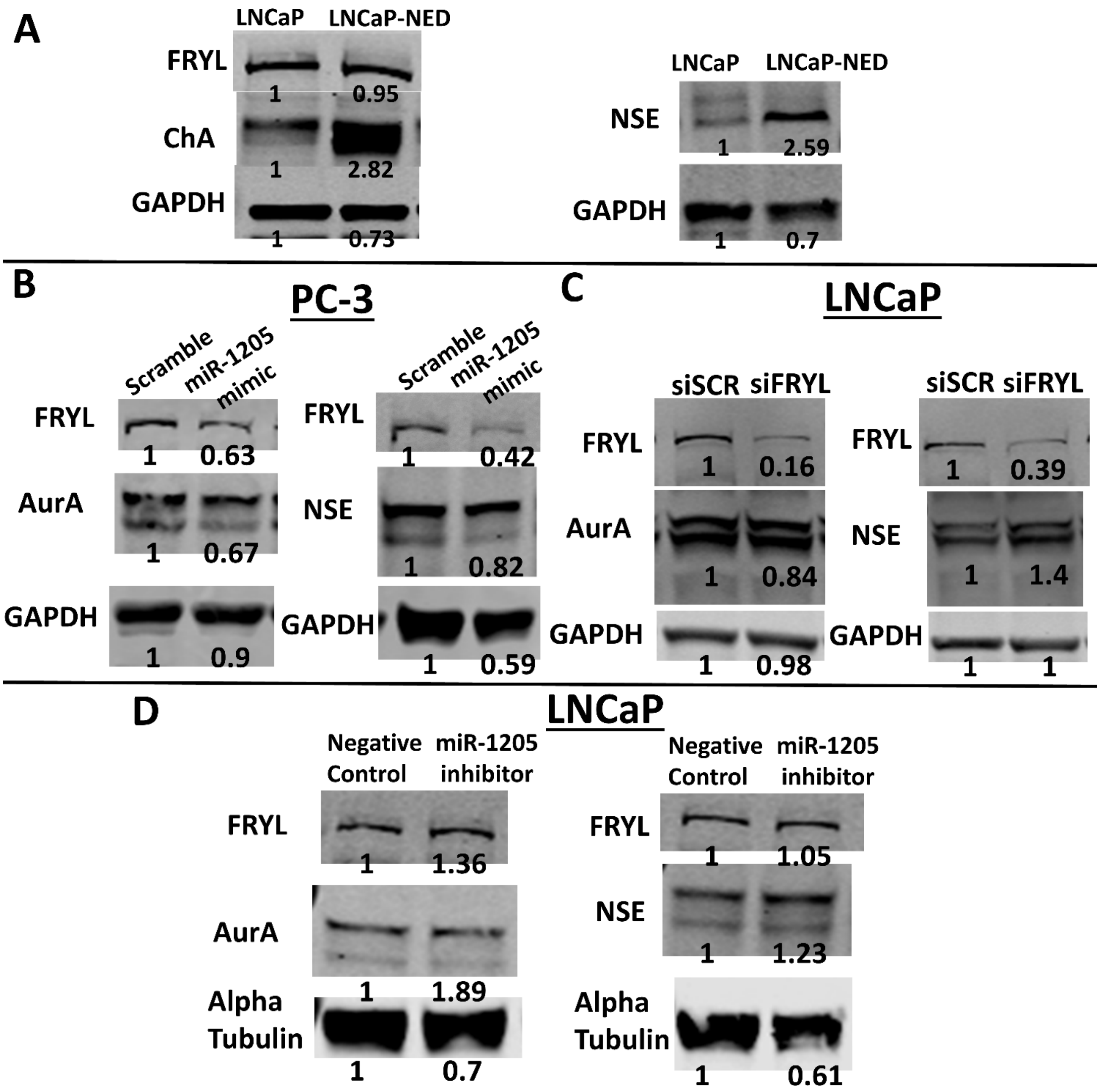

Supplementary Figure 5. miR-1205 regulation of FRYL mRNA may play a role in PCa NED development. (a) Analysis of NED markers (chromogranin A and NSE) and FRYL expression in LNCaP cells after inducing NED by culturing androgen-sensitive LNCaP cells under androgen deprivation conditions. Quantifications were performed using ImageJ analysis normalized to LNCaP cells. (b) A negative control scramble oligonuceltide and miR-1205 mimic were transfected in PC-3 cells to assess induction of NED in androgen-sensitive cells. MiR-1205 overexpression led to a decrease in Aurora A protein expression. Quantifications were performed using ImageJ analysis normalized to Scramble. (c) NSE and Aurora A levels after siRNA-mediated silencing of FRYL in LNCaP cells. Quantifications were performed using ImageJ analysis normalized to siScramble (siSCR). (d) Induction of NED through NSE and AURKA (AurA) overexpression was observed

when LNCaP cells were transfected with a miR-1205 inhibitor. Quantifications were performed using ImageJ analysis normalized to Negative Control.
