# Supplementary material for: MicroRNA-1205 Regulation of FRYL in Prostate Cancer
Source: Front Cell Dev Biol. 2021 Jul 27;9:647485. doi: 10.3389/fcell.2021.647485 (PMC8354587; doi:10.3389/fcell.2021.647485)
Supplement: Supplementary file 2 [file Data_Sheet_2.PDF]

Title:

MicroRNA-1205 regulation of FRYL in prostate cancer

Authors:

Michelle Naidoo[1,2], Fayola Levine[1], Tamara Gillot[1], Akintunde T. Orunmuyi[3], E. Oluwabunmi Olapade-Olaopa[4], Thahmina Ali[1], Konstantinos Krampis[1], Chun Pan[5], Princesca Dorsaint[6], Andrea Sboner[6], Olorunseun O. Ogunwobi[1,2,7]

Affiliations:

[1]Department of Biological Sciences, Hunter College of the City University of New York, New York, NY, USA

[2]Departments of Biology and Biochemistry, The Graduate Center of the City University of New York, New York, NY, USA

[3]Department of Radiation Oncology, College of Medicine, University of Ibadan, Nigeria

[4]Department of Surgery, College of Medicine, University of Ibadan, Nigeria

[5]Department of Mathematics and Statistics, Hunter College of the City University of New York, New York, NY, USA

[6]Englander Institute for Precision Medicine, Weill Cornell Medicine, Cornell University, USA

[7]Joan and Sanford I. Weill Department of Medicine, Weill Cornell Medicine, Cornell University, USA

Corresponding Author:

Olorunseun O. Ogunwobi, MD, PhD

Belfer Research Building

413 E 69th Street, Room 426,

New York, NY 10021, USA

Tel: 212-896-0447; Email: ogunwobi@genectr.hunter.cuny.edu

Keywords: Prostate cancer, FRYL, MicroRNA-1205, neuroendocrine prostate cancer, neuroendocrine differentiation

List of non-standard abbreviations:

PCa, Prostate Cancer; CRPC, Castration-Resistant Prostate Cancer; miR-1205, microRNA-1205; FRYL, Fry-like; AR, Androgen Receptor; ADT, androgen deprivation therapy.

Declaration of interests: The authors declare no competing interests.

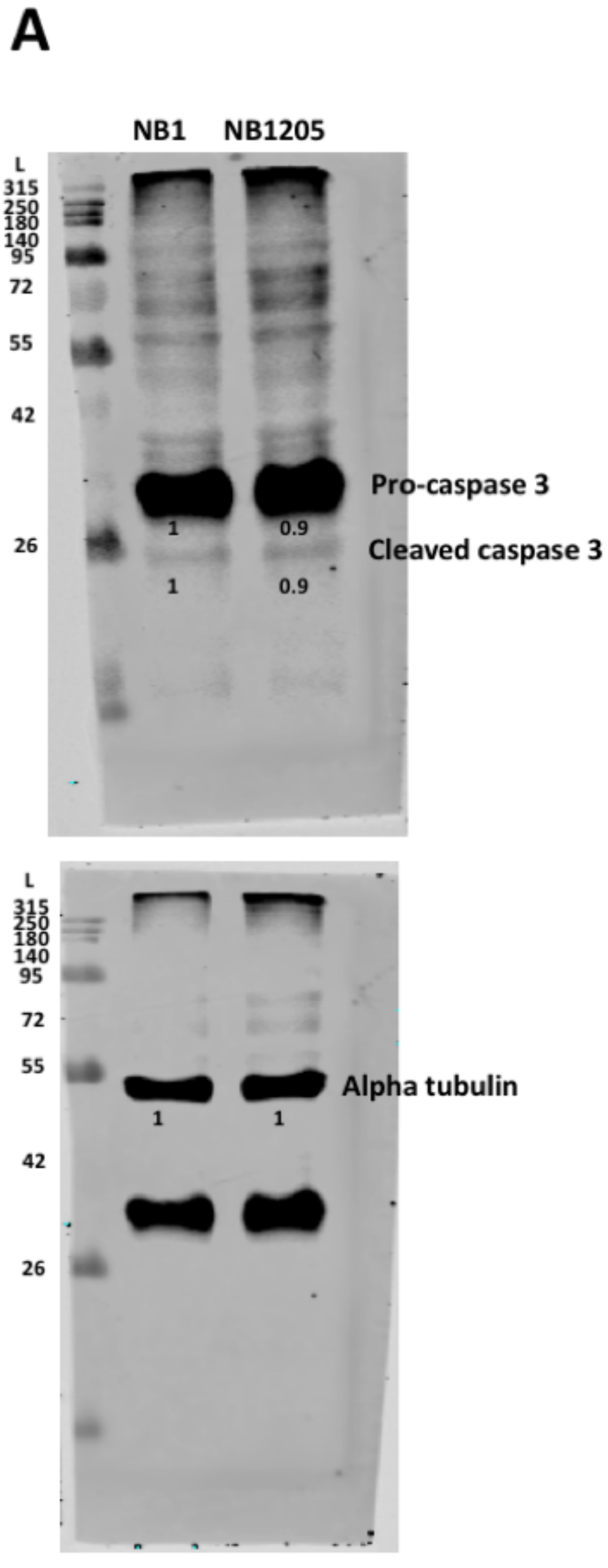
A
NB1
NB1205
L
315
250
180
140
95
72
55
42
26
Pro-caspase 3
1
0.9
Cleaved caspase 3
1
0.9
L
315
250
180
140
95
72
55
Alpha tubulin
1
1
42
26
B
NB1
NB1205
L
315
250
180
140
95
72
Pro-caspase 9
55
1
1.5
Cleaved caspase 9
42
1
1.6
26
L
315
250
180
140
95
72
55
Alpha tubulin
1
1.1
42
26

C

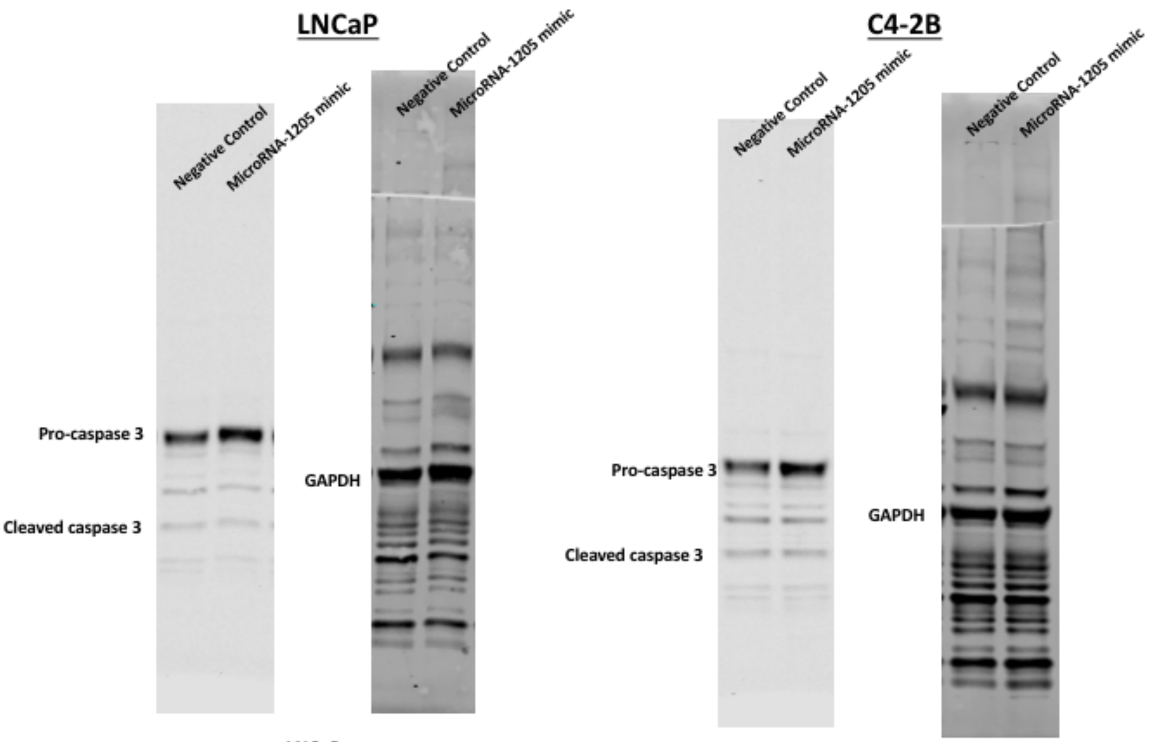
LNCaP
Negative Control
MicroRNA-1205 mimic
Pro-caspase 3
Cleaved caspase 3
GAPDH
C4-2B
Negative Control
MicroRNA-1205 mimic
Pro-caspase 3
Cleaved caspase 3
GAPDH

D

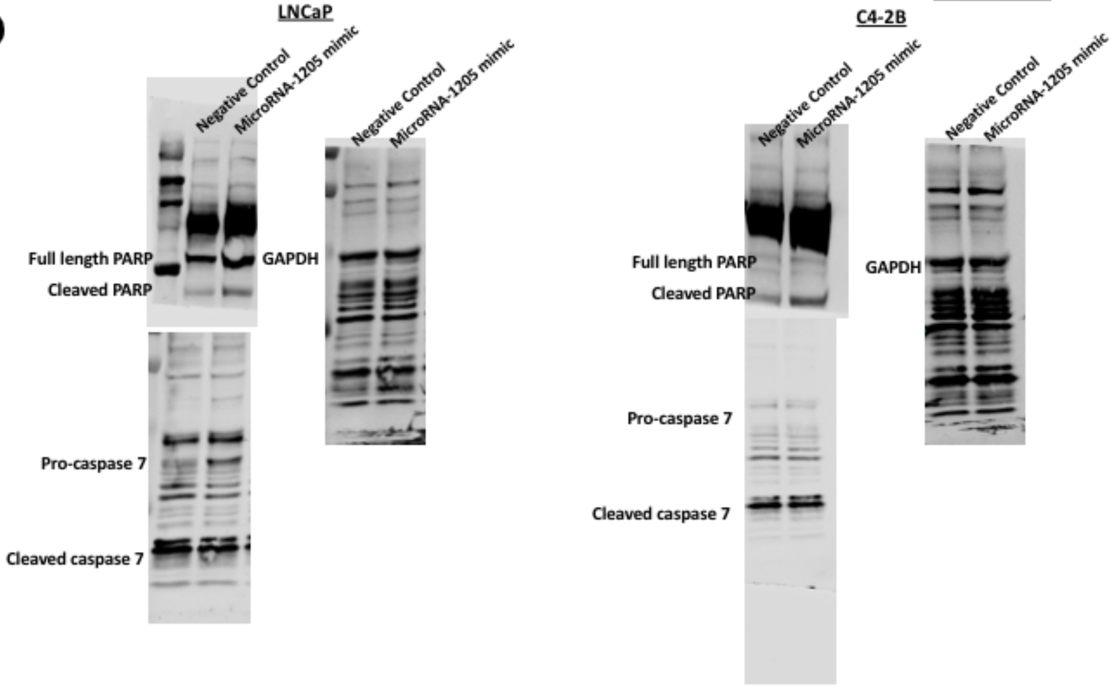
LNCaP
Negative Control
MicroRNA-1205 mimic
Full length PARP
Cleaved PARP
GAPDH
Pro-caspase 7
Cleaved caspase 7
C4-2B
Negative Control
MicroRNA-1205 mimic
Full length PARP
Cleaved PARP
GAPDH
Pro-caspase 7
Cleaved caspase 7

Full western blots for Figure 2 and Supplementary Figure 2. a) Protein cell lysate from C4-2B cells trasnfected with NB1 and NB1205 was analyzed via western blotting. Anti-Caspase 3 was first probed and detected at ~35kDa and its cleaved fragment at ~19kDa (upper blot). The blot was re-probed with anti-Alpha tubulin detected at ~55 kDa as a loading control (lower blot). b) Anti-Caspase 9 was first probed and detected at ~60 kDa (predicted is ~46 kDa) and its cleaved fragment at ~48kDa (predicted is ~37 kDa) (upper blot). The blot was re-probed with anti-Alpha tubulin detected at ~55 kDa as a loading control (lower blot). c) LNCaP and C4-2B cells were transfected with a scramble negative control and miR-1205 duplex. Anti-Caspase 3 was probed and detected at ~35kDa and its cleaved fragment at ~19kDa. The blot was re-probed with anti-GAPDH detected at ~36 kDa as a loading control. d) LNCaP and C4-2B cells were transfected with a scramble negative control and miR-1205 duplex. Anti-Caspase 7 was probed and detected at ~34-35kDa and its cleaved fragment at ~20kDa. PARP and cleaved PARP was detected at 116kDa and 89kDDa, respectively. The blot was re-probed with anti-GAPDH detected at ~36 kDa as a loading control. All quantifications were performed using ImageJ analysis.

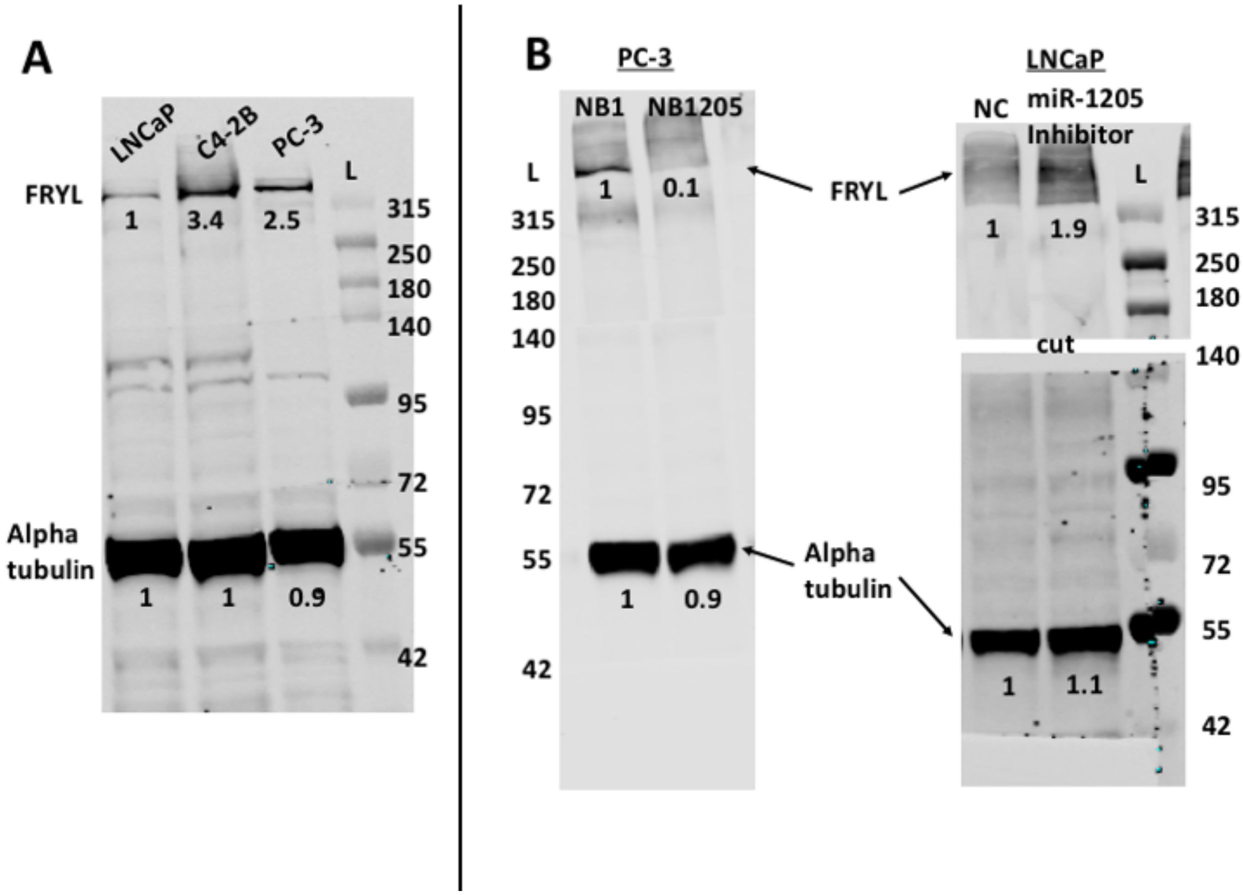

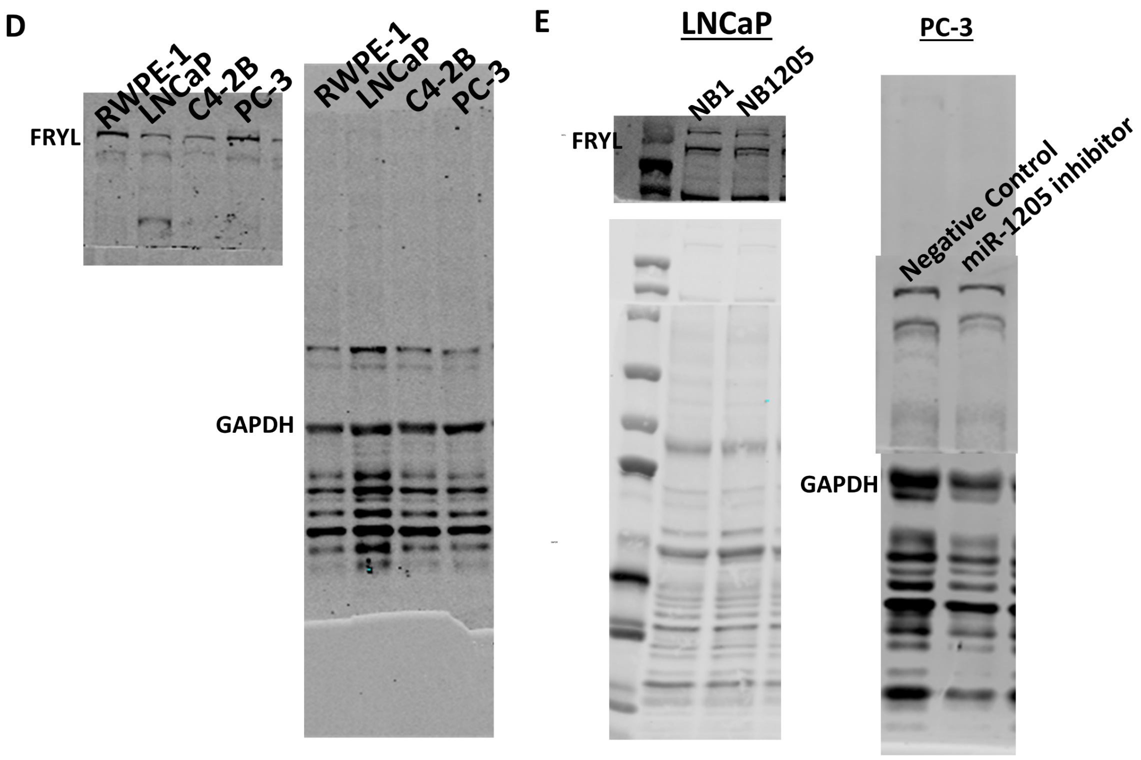

Full western blots for Figure and Supplementary Figure 3. A) Cell lysates from LNCaP, C4-2B and PC-3 cells were analyzed via western blotting. Anti-FRYL was probed and detected at ~340 kDa in LNCaP, C4-2B and PC-3 cells. Anti-alpha tubulin was probed as a loading control and detected at ~55kDa. Bands were normalized to LNCaP. b) Full western blots using protein cell lysates from PC-3 cells transfected with NB1 and NB1205 and LNCaP cells transfected with a negative control oligonucleotide (NC) and miR-1205 inhibitor. Anti-FRYL was probed and detected at ~340 kDa in PC-3 cells transfected with NB1 and NB1205 and LNCaP cells transfected with a negative control (NC) and miR-1205 inhibitor. Anti-alpha tubulin was probed as a loading control and detected at ~55kDa. Bands were normalized to NB1 and NC. All quantifications were performed using ImageJ analysis. d) FRYL protein expression was assessed in RWPE-1 (normal prostate epithelial cells), LNCaP, C4-2B and PC-3 cells. Anti-FRYL was probed and detected at ~340 kDa via western blotting. Anti-GAPDH was probed as a loading control and detected at ~36kDa. Bands were normalized to RWPE-1. e) LNCaP cells transfected with NB1 and NB1205 expression and PC-3 cells transfected with a negative control oligonucleotide (NC) and miR-1205 inhibitor were analyzed for FRYL protein expression via western blotting. Anti-FRYL was probed and detected at ~340 kDa in LNCaP cells transfected with NB1 and NB1205 and PC-3 cells transfected with a negative control (NC) and miR-1205 inhibitor. Anti-GAPDH was probed as a loading control and detected at ~36kDa. FRYL and GAPDH were normalized to NB1 and NC. All quantifications were performed using ImageJ analysis.

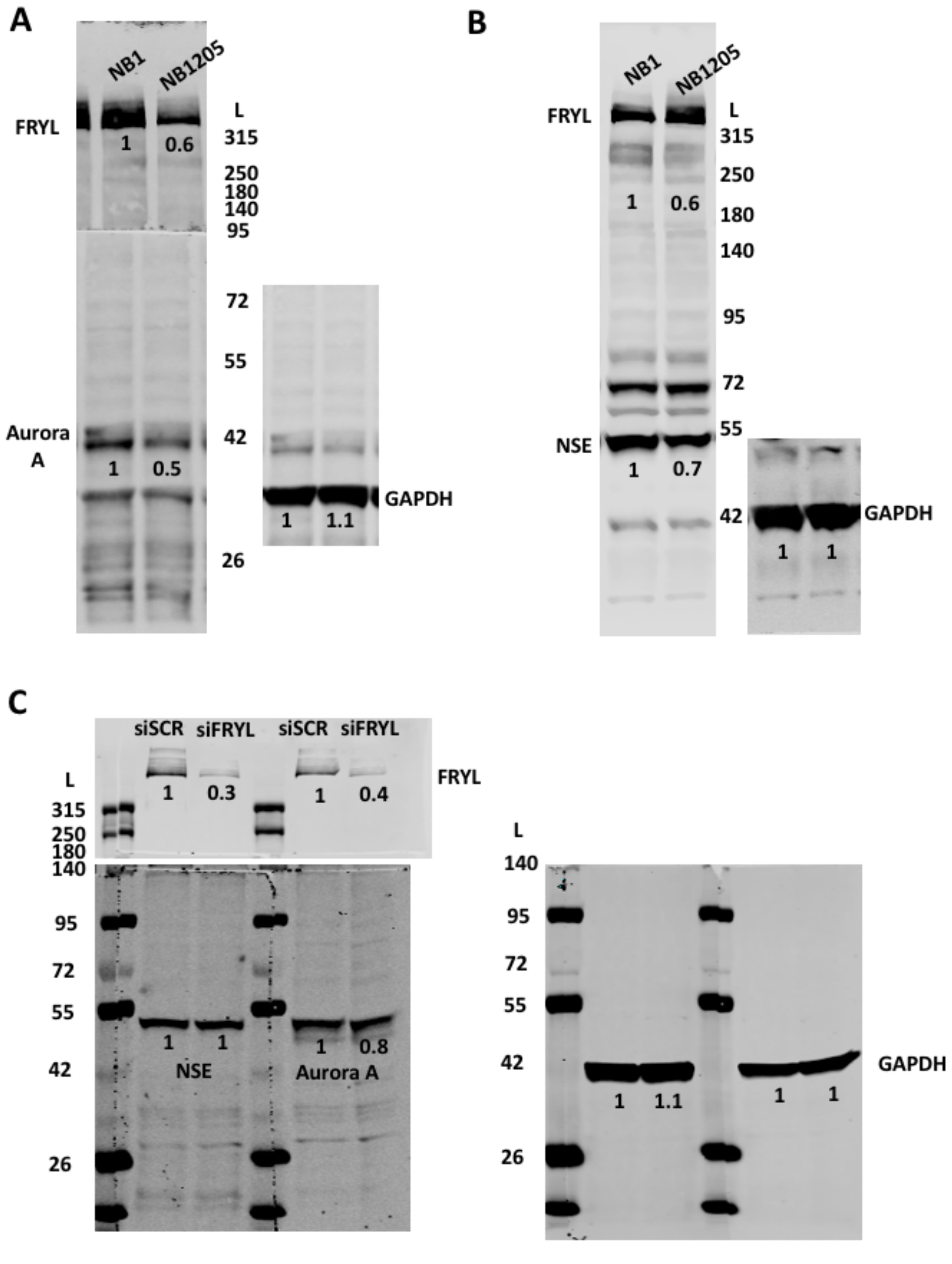

A
NB1
NB1205
FRYL
1
0.6
L
315
250
180
140
95
72
55
Aurora A
1
0.5
42
GAPDH
1
1.1
26
B
NB1
NB1205
FRYL
L
315
250
1
0.6
180
140
95
72
55
NSE
1
0.7
42
GAPDH
1
1
C
siSCR
siFRYL
siSCR
siFRYL
FRYL
L
1
0.3
1
0.4
315
250
180
140
95
72
55
1
1
1
0.8
42
NSE
Aurora A
26
L
140
95
72
55
42
GAPDH
1
1.1
1
1
26

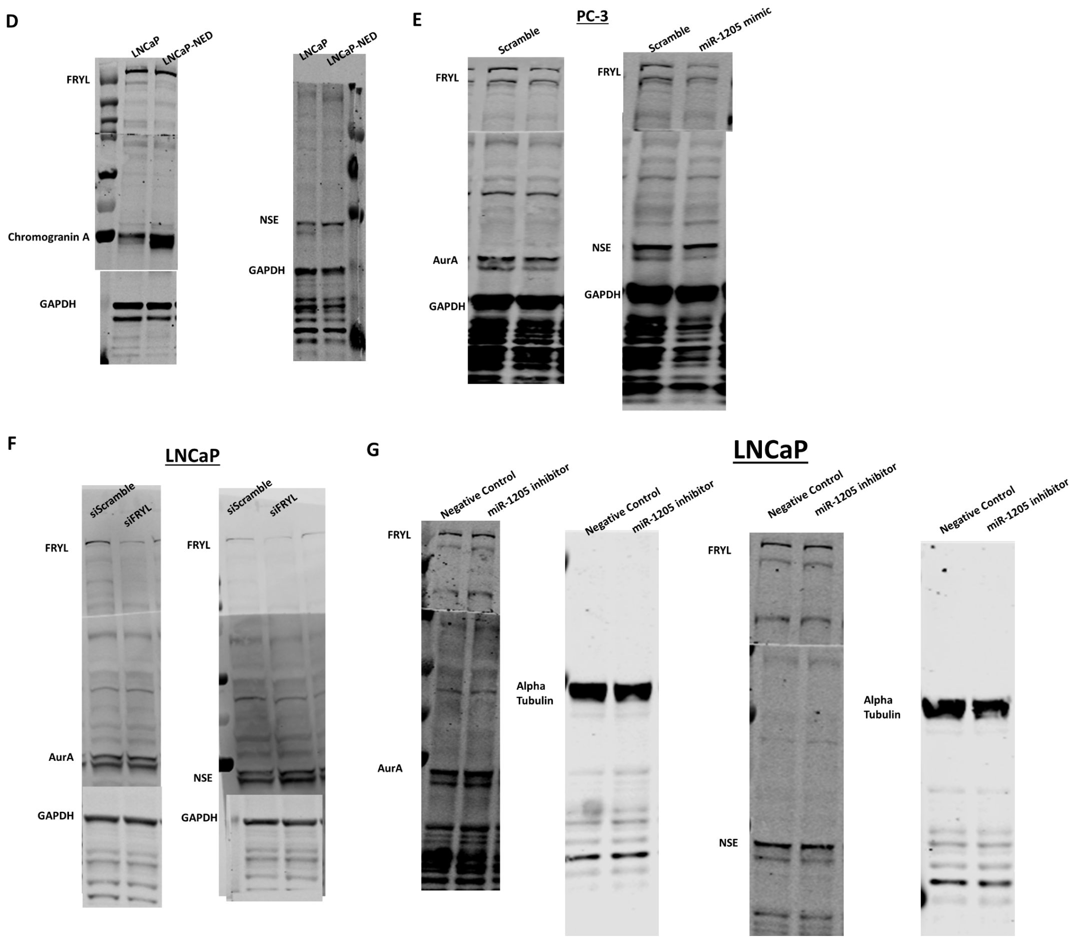

Full blots for Figure and Supplemntary Figure 5. a) Anti-FRYL was probed and detected at ~340 kDa in LNCaP cells transfected with a NB1 (scramble duplex) and NB1205 (miR-1205 duplex). Anti-aurora A was probed and detected at ~48 kDa. Anti-GAPDH was used to re-probe the blot as a loading control (detected at 37 kDa). b) Anti-FRYL was probed and detected at ~340 kDa in LNCaP cells transfected with NB1 and NB1205. Anti-NSE was probed and detected at ~47 kDa. Anti-GAPDH was used to re-probe the blot as a loading control (detected at 37 kDa). c) Anti-FRYL was probed and detected at ~340 kDa in PC-3 cells transfected with an siRNA scramble (siSCR) and siRNA FRYL (siFRYL). Anti-NSE was probed and detected at ~47 kDa (left blot). Anti-aurora A was probed and detected at ~48 kDa (right blot). Anti-GAPDH was used to re-probe the blot as a loading control (detected at 37 kDa). d) Analysis of NED markers (chromogranin A and NSE) and FRYL expression in LNCaP cells after inducing NED by culturing androgen-sensitive LNCaP cells under androgen deprivation conditions. Quantifications were performed using ImageJ analysis normalized to LNCaP cells. (e) A negative control scramble oligonuceltide and miR-1205 mimic were transfected in PC-3 cells to assess induction of NED in androgen-sensitive cells. MiR-1205 overexpression led to a decrease in Aurora A protein expression. Quantifications were performed using ImageJ analysis normalized to Scramble. (f) NSE and Aurora A levels after siRNA-mediated silencing of FRYL in LNCaP cells. Quantifications were performed using ImageJ analysis normalized to siScramble (siSCR). (g) Induction of NED through NSE and AURKA (AurA) overexpression was observed

when LNCaP cells were transfected with a miR-1205 inhibitor. Quantifications were performed using ImageJ analysis normalized to Negative Control.
